# Ligand‐Triggered Self‐Assembly of Flexible Carbon Dot Nanoribbons for Optoelectronic Memristor Devices and Neuromorphic Computing

**DOI:** 10.1002/advs.202207688

**Published:** 2023-02-20

**Authors:** Lin Ai, Yifei Pei, Ziqi Song, Xue Yong, Haoqiang Song, Gongjie Liu, Mingjun Nie, Geoffrey I. N. Waterhouse, Xiaobing Yan, Siyu Lu

**Affiliations:** ^1^ Green Catalysis Center, and College of Chemistry Zhengzhou University Zhengzhou 450000 China; ^2^ Key Laboratory of Brain‐Like Neuromorphic Devices and Systems of Hebei Province College of Physics Science & Technology Hebei University Baoding 071002 China; ^3^ Department of Chemistry University of Sheffield Sheffield S3 7HF UK; ^4^ School of Chemical Sciences The University of Auckland Auckland 1142 New Zealand

**Keywords:** 2D nanoribbons, carbon dots, flexible electronic device, memristor, self‐assembly

## Abstract

Carbon dots (CDs) are widely utilized in sensing, energy storage, and catalysis due to their excellent optical, electrical and semiconducting properties. However, attempts to optimize their optoelectronic performance through high‐order manipulation have met with little success to date. In this study, through efficient packing of individual CDs in two‐dimensions, the synthesis of flexible CDs ribbons is demonstrated technically. Electron microscopies and molecular dynamics simulations, show the assembly of CDs into ribbons results from the tripartite balance of *π*–*π* attractions, hydrogen bonding, and halogen bonding forces provided by the superficial ligands. The obtained ribbons are flexible and show excellent stability against UV irradiation and heating. CDs ribbons offer outstanding performance as active layer material in transparent flexible memristors, with the developed devices providing excellent data storage, retention capabilities, and fast optoelectronic responses. A memristor device with a thickness of 8 µm shows good data retention capability even after 10^4^ cycles of bending. Furthermore, the device functions effectively as a neuromorphic computing system with integrated storage and computation capabilities, with the response speed of the device being less than 5.5 ns. These properties create an optoelectronic memristor with rapid Chinese character learning capability. This work lays the foundation for wearable artificial intelligence.

## Graphical abstract

Self‐assembly of CDs into well‐aligned nanoribbons as active layer materials for memristor applications is demonstrated first time. Flexible memristors (thin as 8 µm) show excellent data storage, retention capabilities (30 days), and fast responses (5.5 ns). The device can simulate biological vision system for Chinese character learning competing to human brains, which lays the foundation for wearable artificial intelligence.

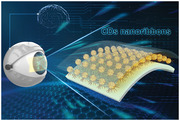

## 1 Introduction

Over the past few years, the development of memristive devices has advanced quickly, inspired by Leon Chua's pioneering theoretical concept of the “memristor” and the first experimental implementation.^[^ ^1^, ^2^ ^]^ Nowadays, memristors are vital electronic components for brain‐inspired neuromorphic computing. Among materials currently under development for constructing memristive systems, which include metal oxides, semiconductors, organic molecules, and low‐dimensional nanomaterials, 2D materials also have been widely studied due to their outstanding electrical tunability, low‐power‐switching capacity, and heterointegration compatibility, properties missing in many conventional bulk 3D materials.^[^ ^3^ ^]^ 2D layered materials, including graphene, transition metal dichalcogenides, and hexagonal boron nitride, have attracted huge attention as promising material platforms for development of high‐efficiency memristors.^[^ ^4^, ^5^ ^]^ For example, a memristor crossbar array was demonstrated using wafer‐scale (2 in.) polycrystalline 2D HfSe_2_ grown by molecular beam epitaxy in combination with a metal‐assisted van der Waals transfer technique, which simultaneously delivered synaptic weight plasticity.^[^ ^6^ ^]^ Yan and co‐workers demonstrated high‐performance and low‐power consumption memristors based on 2D WS_2_ with a 2H phase. The device showed fast ON (OFF) switching times of 13 ns (14 ns), a low program current of 1 µA in the ON state, and a SET (RESET) energy on the level of femtojoules.^[^ ^7^ ^]^ Frequently employed preparation methods toward 2D materials for memristor devices include chemical vapor deposition, wet chemistry, mechanical exfoliation, amongst others.^[^ ^8^, ^9^ ^]^ However, many 2D materials and 2D fabrication techniques have limitations, such as toxic precursor materials,^[^ ^10^, ^11^ ^]^ demanding fabrications such as high temperature and high pressure,^[^ ^12^ ^]^ incompatibility with flexible supports,^[^ ^3^ ^]^ poor scale‐up performance (meaning production in large quantities is difficult), and poor reproducibility.^[^ ^9^, ^13^ ^]^

Self‐assembly is a powerful approach for engineering complex structures of different dimensionality. The approach relies on synthesizing nanometer‐sized building blocks followed by manipulation of their spatial distribution.^[^ ^14^, ^15^ ^]^ This strategy typically relies on weak interactions, such as electrostatics, hydrogen bonding, dipoles, Van der Waals interactions, and combinations thereof, between the nanosized primitive 0D units to form higher‐level architectures.^[^ ^16^, ^17^ ^]^ Advantages of the self‐assembly approach are numerous. First, assembly reduces the exposed specific surface area of the discrete nanodot building blocks, thereby greatly improving the thermodynamic stability of the nanoparticle system.^[^ ^18^ ^]^ Second, self‐assembly allows regulation of the spatial distribution of assembled nano‐sized primitives, thereby allowing the creation of specific architectures (1D, 2D or 3D) for particular applications.^[^ ^19^ ^]^ Third, self‐assembly allows the formation of flexible 1D and 2D structures which are compatible with polymer‐based substrates for the fabrications of flexible devices.^[^ ^20^ ^]^ Fourth, self‐assembly enables large‐scale production of 1D, 2D and 3D arrays at low temperatures and atmospheric pressure with high reproducibility.^[^ ^21^ ^]^ Finally, self‐supporting films can be created without the need of a template, enabling facile device construction without the need for costly template removal processing steps.^[^ ^22^ ^]^ As a proof‐of‐concept, self‐assembly has recently been successfully applied to create complex architectures from simple building blocks, including metal clusters, semiconductor nanoparticles, perovskite quantum dots, organic molecules, and many others.^[^ ^23^, ^24^, ^25^ ^]^ The self‐assembled structures show enhanced performance relative to their nanosized subunits in catalysis, photoelectric devices, energy storage, and other applications.^[^ ^26^, ^27^ ^]^

Carbon is ubiquitous in nature and essential to all life on earth.^[^ ^28^, ^29^ ^]^ Carbon‐based materials are vital in modern electronics and optoelectronic devices, with 2D graphene‐based materials and 0D carbon dots (CDs) attracting a lot of attention in recent years. CDs possess many useful properties including easy preparation, low cost, high water dispersibility, negligible toxicity, electroconductivity, and tunable photoluminescence emissions, leading to their widespread use in electrocatalysis, optoelectronics, energy‐related applications, and fluorescence sensing.^[^ ^30^, ^31^, ^32^ ^]^ These properties have prompted researchers to explore the potential applications of CDs in memristors.^[^ ^33^, ^34^ ^]^ CDs have already been employed as doping materials to enhance electrode conductivity.^[^ ^33^ ^]^ However, due to the poor quality of CDs films, their use as a functional layer in memristors has been limited to date.^[^ ^33^, ^34^ ^]^ While 2D materials are very suitable for optoelectronics,^[^ ^35^ ^]^ the construction of 2D assemblies of CDs has not been experimentally realized to date, representing a major bottleneck to the wider application of CDs.^[^ ^36^, ^37^ ^]^ The discovery of a simple method for the fabrication of 2D CDs arrays would have great significance for the field of CDs, with obvious benefits including: 1) In optoelectronic applications, nanosized CDs are inferior to mature semiconductors due to the high surface energy, instability and easy oxidation.^[^ ^4^, ^34^ ^]^ Stabilization improvements achieved through assembly of CDs into 2D sheets would enable their utilization as the active layer material for optoelectronic devices,^[^ ^18^, ^38^ ^]^ 2) Self‐assembly methods allow adjustment of the spatial distribution of CDs, allowing optimization of optoelectronic properties and performance,^[^ ^19^ ^]^ and 3) CDs are nontoxic, inexpensive, and able to be produced in large quantities from simple precursors, with their 2D assemblies possessing the same advantages and overcoming the limitations of many traditional 2D materials used optoelectronic devices.^[^ ^10^, ^11^, ^39^, ^40^, ^41^ ^]^ Realizing these advantages requires both kinetic and thermodynamic control over morphology evolution during CDs self‐assembly,^[^ ^42^, ^43^ ^]^ which if achieved could allow the construction of CD‐based memristor devices with outstanding performance.

Herein, we report the exciting discovery of a simple one‐pot method for the self‐assembly of CDs into well‐aligned 2D nanoribbons suitable for memristor applications. CDs were first prepared with specific surface organic ligands, with *π*‐*π* interactions, hydrogen‐halogen bonding and hydrogen bonding between the ligands providing the driving force for ordered self‐assembly. The resulting self‐supporting nanoribbons could be easily obtained in powder form for high‐performance memristor device construction. Owing to the excellent stability of the nanoribbon assemblies, the memristor data retention could reach up to 30 days. The flexibility of the nanoribbons allowed the fabrication of transparent devices as thin as 8 µm. Moreover, CDs devices smoothly performed “
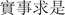
” learning, showed considerable prospects in Chinese character learning. This work establishes important structure–property relationships between the functionality of the individual CDs and the electronic properties of their assemblies, guiding the rational construction of efficient and stable CD‐based memristor devices.

## 2 Results and Discussion

**Figure 1:**
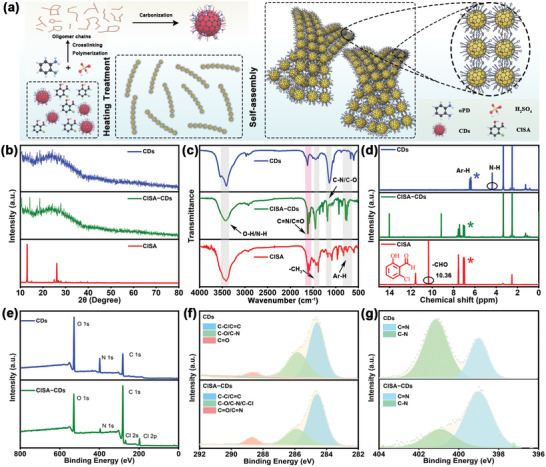
a) Schematic diagram showing the synthesis of the CDs, ClSA‐CDs and ClSA‐CDs nanoribbon assemblies. b) XRD patterns, c) FT‐IR spectra and d) ^1^H NMR spectra of CDs, ClSA‐CDs nanoribbon assemblies and the ClSA ligand. XPS spectra for CDs and ClSA‐CDs assemblies e) XPS survey spectra, f) C 1s spectra, and g) N 1s spectra.

**Figure 2:**
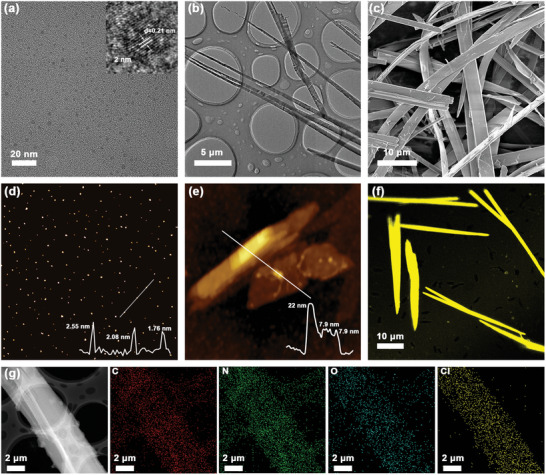
a) TEM, HRTEM (inset), and d) AFM images of pristine CDs. b) TEM, c) SEM, e) AFM, f) confocal fluorescence, and g) HAADF‐STEM and corresponding elemental mapping images of ClSA‐CDs nanoribbon assemblies.

In the present study, the colloidal self‐assembly of aromatic ligand‐decorated CDs was shown to yield well‐defined nanoribbons. The assembly process relied on ligand‐driven inter‐CDs interactions, including *π*–*π* attractions, hydrogen‐bonding and halogen‐bonding interactions (**Figure** **1**a), endowing the nanoribbons with excellent stability and outstanding memristor performance. Firstly, monodisperse CDs (with a diameter of ≈2.65 nm and height of ≈2.55 nm, Figure 2a and Figures S1 and S2d, Supporting Information) were prepared by a simple hydrothermal process from an *o*‐phenylenediamine (oPD) precursor. The obtained CDs possessed abundant amino groups on their surface (Figure 1a).^[^ ^44^ ^]^ High‐resolution transmission electron microscopy (HRTEM) images of the CDs showed lattice fringes with a spacing of 0.21 nm, corresponding to the (100) planes of graphitic carbon (Figure 2a inset), indicating that the carbon dots possessed a crystalline core structure.^[^ ^45^ ^]^ The as‐prepared CDs showed bright excitation‐independent photoluminescence (PL) centered at 600 nm in aqueous solution (Figure S2a, Supporting Information). The construction of 2D CDs assemblies (nanoribbons) required heating the CDs with 6‐chlorosalicylaldehyde (ClSA) capping ligands in ethanol at 80 °C for 2 h in a one‐pot reaction (Figure 1a). After cooling, dialysis purification, and freeze‐drying, it was found that the surface‐modified CDs (that was ClSA‐CDs) had self‐assembled into ultralong nanoribbons (Figures 1a and 2c).

The powder X‐ray diffraction (XRD) patterns (Figure 1b) for the CDs and assemblies were almost identical, showing a broad peak at around 25°. Although the carbon core in the CDs contained some graphitic sp^2^ carbon (as seen by HRTEM), the size of the graphitic domains was very small, thus giving an XRD pattern resembling amorphous sp^3^ carbon.^[^ ^44^ ^]^ The assemblies showed small additional sharp peaks (Figure 1b), indicating that the surface ClSA ligands existed in crystalline domains on the surface of the carbonized carbon core. After functionalization of the CDs with the salicylaldehyde‐based ClSA ligands, some Fourier transform infrared (FT‐IR) spectroscopy peaks appeared at 3420, 1610, and 740 cm^−1^, which could readily be assigned to O—H stretching, C=N stretching and Ar–H vibrations (Figure 1c) of the ClSA ligands, confirming that the ligands were successfully anchored to the carbon core. ^1^H nuclear magnetic resonance (^1^H NMR) measurements (Figure 1d) in dimethylsulfoxide‐d_6_ showed that assemblies possessed more protons in aromatic environments compared to the pristine CDs, was expected since the ligands contained aromatic rings. The aldehyde protons characteristic of the ligands disappeared after reactions with the CDs, confirming the ligand binding involved Schiff base reactions between the ligand aldehyde group and —NH_2_ groups on the surface of the CDs, leading to C=N bond formation (Figure 1d,g). X‐ray photoelectron spectroscopy (XPS) data (Figure 1e) showed that the CDs and the ClSA‐CDs assemblies contained C, N, and O, with the assemblies also containing Cl from the surface ligands. Deconvolution of the high‐resolution N 1s and C 1s XPS envelopes (Figure 1f,g) revealed the presence of more C=N groups in the assemblies, consistent with ligand binding to the carbon core occurring via C=N bonds.^[^ ^46^ ^]^ The relative atomic compositions and functional group speciation determined by XPS (Figure 1e–g and Figure S3, Supporting Information) for the samples are summarized in Table S1 (Supporting Information). Functionalization of the CDs with ClSA ligands and subsequent self‐assembly had a dramatic effect on the optical properties of the CDs. The emission wavelengths of the pristine CDs in ethanol were largely independent of the excitation wavelength (Figure S2a, Supporting Information), whereas the emissions of the ClSA‐CDs in ethanol showed a strong excitation dependence (Figure S2b, Supporting Information). The ClSA‐CDs showed a bright orange‐yellow emission in the solid state (Figure S2c, Supporting Information). Results confirm that the ligand grafting and assembly process modified the electronic energy levels in the CDs.^[^ ^47^ ^]^

The morphology of the ClSA‐CDs nanoribbon assemblies was examined by transmission electron microscopy (TEM) and scanning electron microscopy (SEM). As shown in Figure 2b,c and Figure S4a (Supporting Information), the nanoribbons had widths around 1–3 µm and lengths exceeding 50 µm in some cases. Unfortunately, the individual CDs inside nanoribbons could not be imaged due to the low contrast.^[^ ^48^ ^]^ However, high‐resolution TEM (HRTEM) images of the nanoribbons (Figure S4b,c, Supporting Information) showed a lattice fringes with spacings of 0.21 nm, corresponding to the (100) facet of graphite, confirming that the nanoribbons were composed of individual CDs. Tapping‐mode atomic force microscopy (AFM) showed that the thickness of nanoribbons as low as 7.9 nm (**Figure** **2**d,e, white line), corresponding to a double layer of CDs after taking into account the ligand shell thickness (height 0.55 nm for pure ClSA ligand). The relatively thick lamellas contained up to six layers by AFM (Figure 2e). AFM and TEM images at higher magnifications verified the multilayered structure of the nanoribbons (Figure 2e and Figure S4b, Supporting Information). The confocal fluorescence image in Figure 2f showed that the nanoribbons emitted strong yellow fluorescence under 405 nm excitation. The regular ribbon structure filled the entire screen at low magnifications (Figure S4a, Supporting Information). Element distribution mapping by high‐angle annular dark‐field scanning TEM (HAADF‐STEM, Figure 2g) confirmed a uniform distribution of C, N, O, and Cl in the nanoribbons. This verified that the ligands remained bonded on the surface of the CDs in the 2D assemblies.

**Figure 3:**
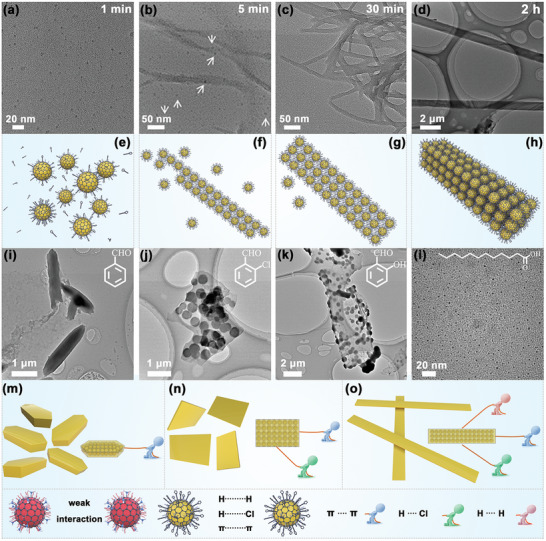
TEM images and schematic evolution of pristine CDs heated with ClSA ligands at 80 °C for a,e) 1 min, b,f) 5 min, c,g) 30 min and d,h) 2 h. TEM images of pristine CDs heated with i) benzaldehyde ligands, j) o‐chlorobenzaldehyde ligands, k) salicylaldehyde ligands or l) dodecanoic acid ligands at 80 °C for 2 h. m–o) Schematic showing the assembled morphologies resulting from different types of inter‐CDs interactions.

The construction of the CDs nanoribbons involved heating the CDs and ClSA in ethanol at 80 °C for 2 h. To gain deeper insights about the self‐assembly process and the morphology evolution of the nanoribbons, intermediates at different stages of the self‐assembly process were collected and characterized (**Figure** **3**a–d). After heating at 80 °C for 1 min in ethanol, the CDs retained their original discrete identity (Figure 3a). Clearly, at short reaction times, the ClSA ligands had not bonded to the CDs to drive the assembly process (Figure 3a,e). On increasing the reaction time to 5 min, 1D‐oriented self‐assemblies of CDs were obtained (Figure 3b). Here, the strong innerlayer interaction between the CDs played a dominant role, controlling the particle orientation and assembly. The 1D structures had a threadlike appearance, with individually unassembled CDs (indicated by white arrows) scattered around the periphery (Figure 3b,f). Even time extension to 10 and 20 min, the single‐particle of CDs was still clearly visible (Figure S5a,b, Supporting Information). HRTEM images revealed clear lattice fringes. The according 0.21 nm distance between the lattice fringes belonged to the (100) plane of graphene sheets (Figure S5c,d, Supporting Information). All these confirmed that the ribbons morphology was assembled via CDs step by step. After heating for 30 min at 80 °C in ethanol, the CDs had started to assemble into 2D arrays of width ≈20 nm and length ≈1 µm (Figure 3c). Heating for 2 h at 80 °C afforded nanoribbons of uniform length, width, and thickness, involving the CDs arranged in multilayered 2D architectures (Figure 3d,h). TEM and SEM revealed that the surface ligands drove the assembly of the CDs to form compact and regular ribbon structures. Heating made the ligands more dynamic and permitted 2D assembly along “y” direction.^[^ ^49^ ^]^ TEM and SEM observations revealed that the final nanoribbons have an average width of about ≈1 µm and lengths extending for tens of microns (Figure 2b,c and Figure S6d, Supporting Information). The SEM study (Figure S6, Supporting Information) showed that with increasing reaction time, the thickness of nanoribbons progressively increased. Clearly, once the assemblies grew to a certain length, van der Waals forces (vdW) between face‐to‐face CDs increased, resulting in lamellar stacking. Thus end‐to‐end assembly of CDs increased the length of the nanoribbons, while the face‐to‐face assembly increased the nanoribbon thickness through the creation of layered structures.^[^ ^49^ ^]^ The multilayered nanoribbons thus utilized at least two types attractions, namely strong inner‐layer and weaker inter‐layer interactions.

**Figure 4:**
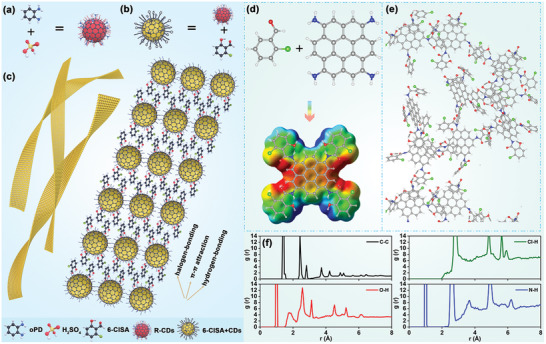
Schematic illustration of the self‐assembly of CDs into well‐defined nanoribbons. The structures of a) pristine CDs, b) ClSA‐CDs, and c) their spatial arrangement within nanoribbons are shown. d) The corresponding model of ClSA‐CDs. e) The 2D‐oriented self‐assembly of ClSA‐CDs. (f) The interaction forces present in the nanoribbon assemblies.

The anisotropy of CDs spatial arrangement was attributed to different forces along the “*x*” and “*y*” directions experienced as the CDs assembled. In the present work, the ligands played a decisive role in this assembly process.^[^ ^49^ ^]^ The main inter‐CDs interactions resulting from dispersion of the ligand‐modified CDs in ethanol would be *π*–*π* attractions between the carbon cores and hydrogen bonding/halogen bonding involving the ligands (**Figure** **4**a–c). Accordingly, we aimed to probe the role of these interactions in the assembly process. First, when the aromatic ligands were replaced by the alkyl chains (dodecanoic acid, DA), no self‐assembly of the CDs was observed (Figure 3l). Clearly, strong inter‐ligand interactions were needed for nanoribbon formation to occur. Second, when ligands with simple unsubstituted aromatic rings were employed as capping ligands, longitudinal assembly of CDs occurred suggesting attraction in a single direction (Figure 3m). A slender rod with a length of about 2 µm was formed (Figure 3i and Figure S7a, Supporting Information), much shorter than the ribbon structures obtained with the ClSA ligand. When a single lateral substituent was added on the aromatic ring in the ligands, lateral hydrogen bonding or halogen bonding occurred, leading to the evolution of wider ribbon‐like structures (Figure 3n). The ribbons obtained possessed a lamellar structure by TEM and SEM, with a ribbon length of about 4–10 µm and a width of about 2–4 µm (Figure 3j,k and Figure S7b, c, Supporting Information). It should be noted that the morphologies of such ribbons determined by TEM were slightly different from those determined by SEM. Clearly, the internal binding force was not strong as that of extended ribbon structures, making the assemblies easily affected by the surrounding environment and sample history. Meanwhile, HRTEM images showed clear (100) lattice plane of graphene (0.21 nm lattice fringe intervals) and the 0.33 nm was attributed to the interlayer spacing in the stacked graphene sheets (Figure S8a–d, Supporting Information), which were matched well with individual CDs (Figure S8e, Supporting Information).^[^ ^48^ ^]^ When using ClSA as the capping ligands, a diverse array of inter‐particle interactions occurred, with the particles assembling in a compact manner to form a strong and stable ribbon structure (Figure 3d,h). Tripartite forces (*π*–*π* attractions, hydrogen bonding and halogen bonding all contributed to the oriented assembly, leading to ribbons that were regular in both width (1–3 µm) and length (extending up to 100 µm) (Figure 3o).

For deeper understanding of the self‐assembly mechanism, we performed a molecular dynamics simulation using the generalized amber force field (GAFF) approach. To simplify the model, short graphite flakes were employed to represent the carbon core in the CDs. To model the pristine CDs, amino groups were added onto the graphite flakes to represent the core‐shell structure. About 50 graphite flakes were used in the simulation, which was found to assembly in a disordered aggregated structure through *π*–*π* attractions between the carbon cores (Figure S9, Supporting Information). Since the actual inner core of the CDs contained both sp^2^ and sp^3^ carbon atoms and abundant functional groups on the surface, repulsive forces made CDs scatter during TEM analyses. When the ClSA ligands were attached to the surface of the graphite flakes through C=N bonds (Figure 4d), the flakes start to assemble into 2D‐like structures (Figure 4e and Figure S10, Supporting Information). The molecular electrostatic potential (MEP) plot for the ClSA‐modified flakes showed alternating electron‐rich and electron‐deficient regions on the surface. This pattern suggested the possibility of long‐range Cl…H, N…H, and O…H interactions in the ribbons (Figure 4f). Further, intra‐plane *π*–*π* interactions were also formed. The C—C radial distribution function (rdf) suggested the flakes have C—C distances around 3.5 Å, with Cl…H, N…H, and O…H interactions at 2.7–3.2 Å assisting with the formation of 2D structures. These results clearly indicate that in addition to a *π*–*π* effect involving the CDs cores, hydrogen bonds and hydrogen‐halogen bonds between ligands also played a key role in nanoribbon formation.

In addition to the ligands, other parameters contributed to the formation to the nanoribbons, including temperature, ligand density and the carbon core. Experiments with ClSA‐CDs at lower temperatures (e.g., 50 °C or room temperature) also resulted in self‐assembled structures (Figure S11, Supporting Information), but their morphologies were much less regular than the nanoribbons obtained at 80 °C in ethanol. We hypothesize that a temperature of 80 °C enhanced aromatic ring mobility in the ClSA ligands, enabling the distribution and reorganization of the aggregated CDs into well‐ordered nanoribbons. Next, reducing the density of the ClSA ligands on the surface the CDs by a large margin yielded disordered structures. Clearly, a critical amount of surface ClSA ligands is needed to drive nanoribbon assembly (Figure S12, Supporting Information). Subsequently, the effect of the carbon core on the self‐assembly process was explored. We used *o*‐phenylenediamine (oPD) and citric acid (CA) as the reaction precursors, together with two‐step grafting ligands to prepare CDs2. The sample had few amino groups for anchoring ClSA ligands, thus weaker *π*–*π* attractions between CDs. Further, the sample had more carboxyl groups for hydrogen bonding. In the presence of ClSA ligands, short flakes rather than nanoribbons were obtained (Figure S13a,b, Supporting Information). When ethylenediamine was utilized to replace oPD to form CDs3, the carbon core in the obtained CDs lacked sufficient rigidity to allow orientated assembly, resulting in the formation of irregular spheres rather than nanoribbons (Figure S13c,d, Supporting Information). Results show that both the central carbon core and the abundance of surface ligands affected the morphology of the assemblies. It should be noted that the pure ClSA ligands did not form 2D ribbons (Figure S14, Supporting Information), eliminating the possibility that ligand residues were responsible for the structures observed by SEM and TEM for the ClSA‐CDs assemblies.

Further tests showed the assembled ClSA‐CDs nanoribbons to possess outstanding stability. For the small size and high specific surface area made the single CDs unstable and tended to agglomerate, while 2D assembly was more stable with the reducing specific surface area. Meanwhile, the surface of CDs here was coated with ligands, and the ligands were entangled on the surface during the assembly process to form a stable morphology. The surface ligands could effectively prevent the direct erosion of water and oxygen on the carbon core, so the stability could be greatly improved.^[^ ^47^, ^49^ ^]^ UV irradiation (365 nm) for 36 h had no effect on the nanoribbons (Figure S15, Supporting Information). Further, heating the materials in an oven for different time periods left the morphology and optical properties of the nanoribbons practically unchanged (Figure S16, Supporting Information). These experiments demonstrate that the nanoribbons were stable to UV and heat, making them very suitable for application in devices. The 2D nanoribbon morphology was preserved even after one year under ambient conditions (Figure S17, Supporting Information).

**Figure 5:**
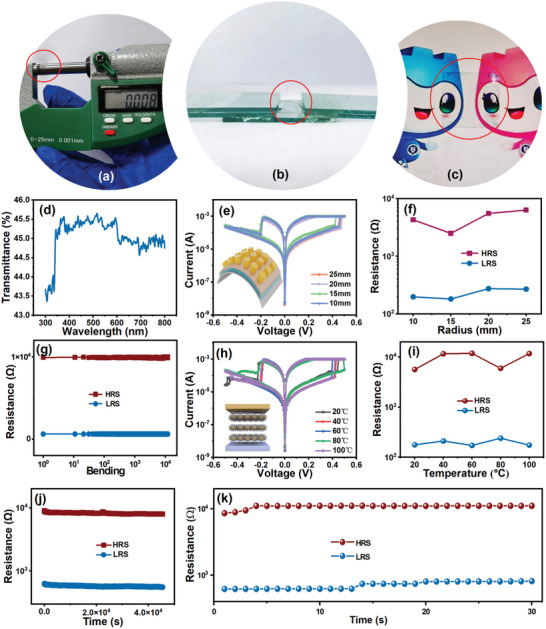
a) Photograph showing the thickness of memristor with the TiN/ClSA‐CDs/ITO/mica construction was 8 µm. b) Photograph of the TiN/ClSA‐CDs/ITO/mica memristor device with a thickness of ≈8 µm after bending. c) Photograph showing the high optical transparency of the TiN/ClSA‐CDs/ITO/mica memristor device in (b). d) UV–vis transmittance spectrum of the TiN/ClSA‐CDs/ITO/mica memristor device. e) *I*–*V* curves as a function of the bending radius. f) Statistics of HRS and LRS of TiN/ClSA‐CDs/ITO/mica memristor device at different bending degrees. g) Resistances of the HRS and the LRS under different bending configurations. h) *I*–*V* sweep curves at different temperatures from 20 to 100 °C. i) Statistics of HRS and LRS of the TiN/ClSA‐CDs/ITO/mica memristor device from 20 to 100 °C. j) The retention characteristics of the device at room temperature. k) Resistance states of the device over 30 consecutive days without applying a voltage.

A flexible transparent memristor was subsequently prepared using the ClSA‐CDs nanoribbons, with the electrical characteristics of the CDs‐based memristor shown in **Figure** **5** (the manufacturing details of CDs memristor devices are described in the Experimental Section). Here, we focused on both the storage capability of the memristor and also its flexibility for use in wearable devices. Compared with traditional memristor substrates, mica substrates show good mechanical flexibility, leading to their use in this work. TiN/ClSA‐CDs/ITO/mica memristor devices were constructed in this work with a thickness ≈8 µm (Figure 5a). The ITO and TiN layers were deposited by magnetron sputtering, whilst the ClSA‐CDs layer was deposited by spin‐coating. If the ClSA CDs aqueous solution was directly used for spin coating, the uniformity of the film may be poor. However, we configured ClSA CDs: pss solution through process exploration to ensure proper viscosity, and chose 3000 rpm speed and 80 °C drying temperature to ensure the uniformity of film formation. Figure S18 (Supporting Information) showed the SEM images of ClSA CDs aqueous solution and pss solution respectively. It can be found that ClSA CDs were uniformly embedded in pss and ClSA CDs: pss film had a small roughness. The ClSA‐CDs memristor devices also exhibited great flexibility and extensive bending properties as shown in Figure 5b. The bending length (2R) was 10 mm (original length was 15 mm), and the thickness (*t*) was 0.008 mm (mica substrate and film included). The film's strain value was calculated as 0.0008 according to the formula:^[^ ^50^ ^]^

(1) $$\varepsilon =\frac{t}{2R}\;$$

TiN and ITO are promising materials for next‐generation transparent conducting electrodes,^[^ ^51^ ^]^ possessing high optical transparency at visible wavelengths. Accordingly, the assembled TiN/ClSA‐CDs/ITO/mica structure showed excellent optical transparency (Figure 5c). As shown in Figure 5d, the corresponding CDs‐based device showed good optical transparency (≈45.5% in the visible range from 350 to 600 nm). Figure 5e shows *I*–*V* curves collected for the ClSA‐CDs memristor device at various bending radii, with the response at different degrees of bending showing little variation. Further, the resistances of the high resistance state (HRS) and low resistance state (LRS) did not exhibit any apparent oscillations (Figure 5f). Even after bending the ClSA‐CDs flexible memristor device 10^4^ times, the resistances in the HRS and LRS maintained a decent ratio and were remarkably distinct (Figure 5g). Results suggest the device has potential for application in wearable electronics. However, the pristine carbon dots without self‐assembly have low film homogeneity in the memristor device, which has no obvious resistance‐switching characteristics (Figure S19, Supporting Information). Figure S20 (Supporting Information) showed the distribution of ClSA‐CDs in the device. However, other ligands formed a thin rod structure with a length of about 2 um, much shorter than the ribbon structures obtained with the ClSA ligand. Figure S21a–d (Supporting Information) showed the dispersion of benzaldehyde ligands, o‐chlorobenzaldehyde ligands, salicylaldehyde ligands, and dodecanoic acid ligands capped CDs in the device under the same experimental conditions. Figure S21e–h (Supporting Information) showed the corresponding device performance. It can be found that the device has short circuit and small window, which is not suitable for memristors. High film homogeneity is important for large‐scale integrated circuits, providing a basis for future work. To exclude the effect of ligands, we found no significant resistance‐switching effect in the ligand devices prepared under the same experimental conditions, as shown in Figure S22 (Supporting Information). The ClSA‐CDs nanoribbons possess the desirable characteristics of high‐temperature resistance and strong adhesion.^[^ ^52^ ^]^ Impressively, the *I*–*V* curve for the memristor device containing ClSA‐CDs did not fluctuate significantly in the range 20–100 °C, as shown in Figure 5h, suggesting suitability for application in wearable electronics. Figure 5i showed the HRS and LRS of the ClSA‐CDs memristor device at different temperatures. The device showed good stability over the entire temperature range. The retention qualities of a memristor device reflect its capacity to store and maintain data once it has been written.^[^ ^53^ ^]^ Figure 5j shows the retention properties of the ClSA‐CDs memristor device, which has no distinct resistance degeneration until 4 × 10^4^ s. We removed the test voltage and found that ClSA‐CDs memristor device could maintain its HRS and LRS for 30 d as shown in Figure 5k. The device also shows good uniformity, Figure S23 (Supporting Information) analyzes the statistical data of the Gaussian fitting histogram of the threshold voltage of the same device. The threshold voltage value is about 0.46 and ‐0.20 V respectively. Figure S24 (Supporting Information) shows the threshold voltage distribution of five randomly selected devices. The variation [*δ* = Δ*V*(2*σ*)/*V*_mean_] in the SET and RESRT voltages of all of these devices are less than 5%. Table S2 (Supporting Information) summarizes the performance of 2D material memristors reported in recent years. The comparison reveals that the ClSA‐CDs memristor device offered outstanding stability and bending durability, laying a material and device foundation for wearable artificial intelligence.

**Figure 6:**
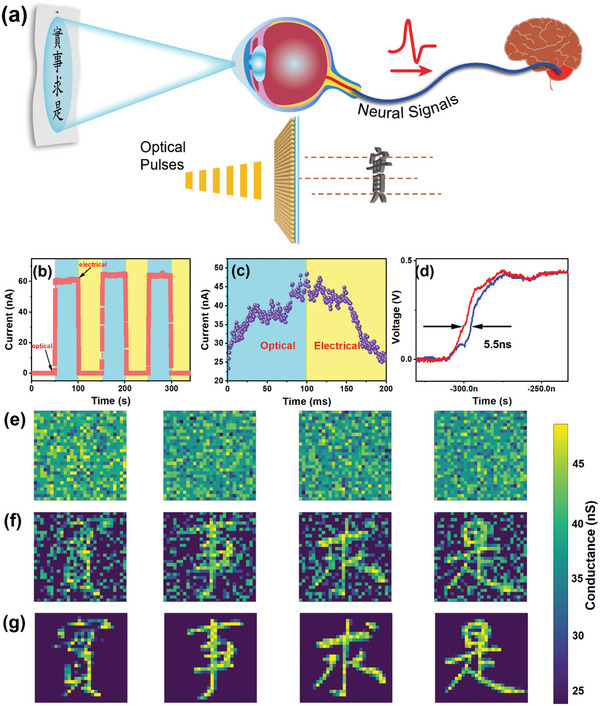
a) Schematic diagrams of a learning task involving the human vision system and also a CDs‐based memristor device. b) Pulse switching characteristics of a ClSA‐CDs memristor device under chopped light. c) Light‐intensity‐dependent LTP with a pulse width of 1 ms. d) The response speed of ClSA‐CDs memristor device can reach 5.5 ns. e–g) Chinese character image memory demonstration.

The physical separation of data storage and processing units is the foundation of traditional computing designs, which leads to computers having low computational efficiencies. Accordingly, the development of highly efficient neuromorphic computing systems with integrated storage and computation capabilities has captured the imagination of scientists.^[^ ^54^ ^]^ It is possible to build an artificial visual system for real‐time in‐sensor computing using optoelectronic memristors, which exhibit great promise in the integration of sensing, memory, and computing functions.^[^ ^55^ ^]^ Due to their strong light–matter interactions and effective photogenerated charge trapping arising from their extremely high surface‐to‐volume ratio, 2D materials and their hybrid heterostructures represent suitable platforms for nonvolatile photonic memory devices.^[^ ^56^ ^]^ The intrinsic optical properties and light‐responsiveness of CDs create opportunities for artificial vision systems utilizing CDs‐based optoelectronic memristors.^[^ ^57^ ^]^ Optoelectronic memristors combine the benefits of a photodetector and electrical resistive random access memory (RRAM) for real‐time perception and nonvolatile storage. **Figure** **6**a depicts an optoelectronic memristor for learning Chinese characters, in which optical stimuli for a Chinese character was input directly into a signal processing system without the use of special image sensors or associated analog‐to‐digital data conversion. By comparing the response currents of a perfect device with and without illumination, the photoresponse characteristics of the device can be confirmed. Figure 6b shows that the current for a memristor based on a TiN/ClSA‐CDs/ITO/mica films suddenly increased when a 405 nm light pulse (the assembled materials had a matching absorption at 405 nm) was applied to the optoelectronic device, allowing the perception of light information, and then gradually decayed when the light was removed. To better explore the device photostimulation turn‐on process, we stimulated the ClSA‐CDs memristors device with smaller pulse width light pulses, as shown in Figure 6c. The device can control the resistance value through optical and electrical signals, so as to design continuous optical and electrical waveforms, which can simulate the enhancement and inhibition behavior of artificial synapses. The conductance state of the memristor increased during optical modulation and decreased during electrical modulation, corresponding to the long‐term potentiation (LTP) and long‐term depression (LTD) properties. The weight change in brain‐like neuromorphic computing was realized by optoelectronic LTP/LTD, which operated at a speed of 1 ms for optical stimulation, which was faster than the level of the human brain. We apply different number of optical pulses to make the device reach different states, then remove the light, only keep the reading voltage applied to the device to read the current value of the device. Figure S25 (Supporting Information) shows the intermediate state of attenuation to the interior after removing the optical pulse after continuously applying 25, 50, 75, and 100 continuous optical pulses to the device. By comparing the current values of the initial state and the intermediate state, it is shown that the short‐term potentiation (STP) can be simulated after 25 optical pulses, and the long‐term potentiation (LTP) can be simulated after 50, 75, and 100 optical pulses. Figure 6d showed the response speed of the device to be only 5.5 ns, which makes it possible to apply the device in the high‐speed artificial vision systems. Table S3 (Supporting Information) shows the advantages of ClSA‐CDs devices compared with other devices for visual learning. Next, was applied the ClSA‐CDs memristors device to learning the Chinese characters “
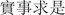
” (which stand for “seek truth from facts” in English). The weight values were randomly initialized in the 28*28 array, with the weight adjustment results displayed of the Chinese characters shown in Figure 6e–g, respectively. Weight distribution after 1, 100, and 200 training iterations are shown in Figure S26 (Supporting Information). The Chinese characters for “seeking truth from facts” were clearly displayed after the 200th iteration. Figure S27 (Supporting Information) showed the distribution of synaptic weights. Results show that the system can fine‐tune synaptic weights, enabling the memristor based on aTiN/ClSA‐CDs/ITO/mica films to learn Chinese characters at high‐speed.

## 3 Discussion

In summary, 2D architectures with controlled ribbons morphology were produced through the self‐assembly of CDs capped with aromatic ligands in ethanol at 80 °C. The main driving forces of such self‐assembly were *π*–*π* interactions and the hydrogen/halogen‐bonding interactions between the capping ligands on the CDs. The strong *π*–*π* interactions initially create 1D‐CDs assemblies, with subsequent hydrogen/halogen‐bonding interactions leading to the attachment of CDs on both sides, leading to 2D structures. These lamellae then overlapped to form thicker nanoribbon‐like structures comprising several layers of CDs. The visualization of self‐assembled structure and dynamics at the nano‐level has become a powerful method to understand structure‐function relationships of self‐assembly. The nanoribbons were then applied as the active layer of a memristor device, which possessed a data storage capacity extending to one month. The device was used to simulate biological vision system and realize rapid learning of Chinese characters. The CD‐based memristor holds promise in flexible wearable devices and artificial neural networks.

## 4 Experimental Section

### Materials

Absolute ethanol (EtOH, G.R.), citric acid (CA), o‐phenylenediamine (oPD), ethylene diamine (EDA), salicylaldehyde (SA), benzaldehyde (BA), 2‐chlorobenzaldehyde (ClBA), 2‐chloro‐6‐hydroxybenzaldehyde (ClSA), and dodecanoic acid were purchased from Shanghai Macklin Biochemical Co., Ltd. Concentrated H_2_SO_4_ (98 wt%) were purchased from Shuangshuang Chemical Co., Ltd. All chemical reagents were used directly as received without further purification. Ultrapure (Milli‐Q) water was used for all experiments and obtained from a SZ‐93A water purification system.

### Synthesis of CDs

oPD (0.108 g, 1 mmol), 1 mL of concentrated H_2_SO_4_, and 10 mL of deionized water were transferred to a 25 mL autoclave and heated at 200 °C for 6 h. After the autoclave had cooled to room temperature, the obtained dispersion was filtered with a filter membrane (0.22 µm), dialyzed for 24 h, then finally freeze‐dried to obtain CDs.

### Synthesis of CDs2

oPD (0.108 g, 1 mmol), CA (0.192 g, 1 mmol) and 10 mL of deionized water were transferred to a 25 mL autoclave and heated at 200 °C for 6 h. After the autoclave had cooled to room temperature, the obtained dispersion was filtered with a filter membrane (0.22 µm), dialyzed for 24 h, and then finally freeze‐dried to obtain CDs2.

### Synthesis of CDs3

CA (0.961 g, 5 mmol), EDA (335 µL, 5 mmol), and 10 mL of deionized water were transferred to a 25 mL autoclave and heated at 200 °C for 5 h. After the autoclave had cooled to room temperature, the obtained dispersion was filtered with a filter membrane (0.22 µm), dialyzed for 24 h, and then finally freeze‐dried to obtain CDs3.

### Self‐Assembly of CDs

CDs (25 mg), ligand (see below), and 20 mL of EtOH (G.R.) were added to a 50 mL round‐bottomed flask and the resulting mixture was heated under reflux at 80 °C for 2 h. After cooling to room temperature, the dispersions were dialyzed for 20 d, then finally freeze‐dried to obtain ClSA‐CDs. The ligands used were ClSA (90 mg, 0.575 mmol), SA (70 mg, 0.573 mmol), BA (60 mg, 0.565 mmol), ClBA (80 mg, 0.569 mmol) or dodecanoic acid (160 mg, 0.566 mmol).

### Characterization

UV–vis absorbance spectra were collected on a LAMBDA 1050 spectrophotometer (PerkinElmer). Fluorescence spectra and lifetimes were measured on an FLS‐1000 spectrofluorometer (PerkinElmer). X‐ray photoelectron spectroscopy (XPS) measurements were performed on a Thermo Fisher ESCALAB 250Xi surface analysis system equipped with an Al K*α* source. X‐ray diffraction (XRD) patterns were acquired on an X'Pert PRO instrument (PANalytcal) over the scan range 5–90° at 3° min^−1^ (Cu‐K*α* radiation, 40 kV, 40 mA, *λ* = 1.5418 Å). The nuclear magnetic resonance (NMR) spectra were recorded on a Bruker 400 NMR spectrometer. Fourier transform infrared spectroscopy (FTIR) analyses used a PerkinElmer Spectrum Two spectrometer. Transmission electron microscopy (TEM) images were collected on a TecnaiG2F20 S‐Twin TMP electron microscope operating at an accelerating voltage of 200 kV. Atomic force microscopy (AFM) images were recorded in tapping mode on a Dimension Icon scanning probe microscope from LabRAM HR Evo under ambient conditions. Scanning electron microscopy (SEM) images were collected on a field emission scanning electron microscope (SEM, Phenom). Fluorescent microscopy was recorded on the LEICA TCS SP8 STED using a STED excitation source for fluorescent images.

### CDs Memristor Device Fabrication

First, an adhesive tape was used to peel a thin mica sheet off a block of mica. Next, the tape with the thin mica sheet was immersed in acetone to obtain a thin free‐standing mica sheet, which was then washed thoroughly with ethanol and water. As the substrate of the device, mica, a layered oxide material with a high melting point of 1300 °C. After drying, a 100 nm thick ITO layer was deposited on one side of the mica substrate by RF magnetron sputtering, then quickly annealed at 500 °C to improve the conductivity and transmittance of the ITO layer. The ITO is used as the bottom electrode, which is transparent in the visible region and displays a high conductivity of 10–10^3^ S cm^‐1^. Subsequently, a ClSA‐CDs layer was spin‐coated onto the ITO layer to form a uniform film (for this, the ClSA‐CDs were dispersed in pss colloid at a concentration of 10 mg mL^‐1^). Finally, using a shadow mask with a diameter of 200 µm, a TiN layer (top electrode layer, about 40 nm thick) was grown by DC magnetron sputtering at a pressure of 0.8 Pa and an Ar flow rate of 25 sccm. The TiN is used as the top electrode, due to the ability to control and adjust the distribution of movable oxygen ions and oxygen vacancies. The ClSA CDs: pss is the functional layer of the device. The obtained flexible CDs‐based memristor device had a thickness of ≈8 µm and a high optical transparency.

### Simulation

Molecular dynamics (MD) simulations as implemented in the large‐scale atomic/molecular massively parallel simulator (LAMMPS)^[^ ^58^ ^]^ were performed to study the growth of CDs. The Amber general force field (GAFF)^[^ ^59^ ^]^ was used. The Antechamber software was employed to generate the topologies for the MD simulations. The atomic charges are represented with the restrained electrostatic potential (RESP)^[^ ^60^ ^]^ which were computed at the HarteeFock level with the 6‐311G** basis set and MerzSinghKollman scheme by using the Gaussian 09 program. ^[^ ^61^ ^]^ The CDs were represented with graphene‐like coronene structures. The ligand is added by forming new C=N bonds. To simulate the growth of CDs, Isothermal‐isobaric (NPT) ensemble simulations were performed for 100 ns on a model contained 200 CDs. A time step of 0.25 fs was employed.

## Conflict of Interest

The authors declare no conflict of interest.

## Supporting information

Supporting information

## Data Availability

The data that support the findings of this study are available from the corresponding author upon reasonable request.
