# Supplementary material for: Ligand‐Triggered Self‐Assembly of Flexible Carbon Dot Nanoribbons for Optoelectronic Memristor Devices and Neuromorphic Computing
Source: Adv Sci (Weinh). 2023 Feb 20;10(12):2207688. doi: 10.1002/advs.202207688 (PMC10131856; doi:10.1002/advs.202207688)
Supplement: Supplementary file 1 — Supporting information [file ADVS-10-2207688-s001.pdf]

ADVANCED SCIENCE
Open Access

Supporting Information

for *Adv. Sci.*, DOI 10.1002/advs.202207688

Ligand-Triggered Self-Assembly of Flexible Carbon Dot Nanoribbons for Optoelectronic Memristor Devices and Neuromorphic Computing

Lin Ai, Yifei Pei, Ziqi Song, Xue Yong, Haoqiang Song, Gongjie Liu, Mingjun Nie, Geoffrey I. N. Waterhouse, Xiaobing Yan and Siyu Lu**

Supporting Information

Ligand-Triggered Self-Assembly of Flexible Carbon Dot Nanoribbons for Optoelectronic Memristor Devices and Neuromorphic Computing

Lin Ai,[1,#] Yifei Pei,[2,#] Ziqi Song,[1,#] Xue Yong,[3] Haoqiang Song,[1] Gongjie Liu,[2] Mingjun Nie,[1] Geoffrey I. N. Waterhouse,[4] Xiaobing Yan,[2,] Siyu Lu[1,*]*

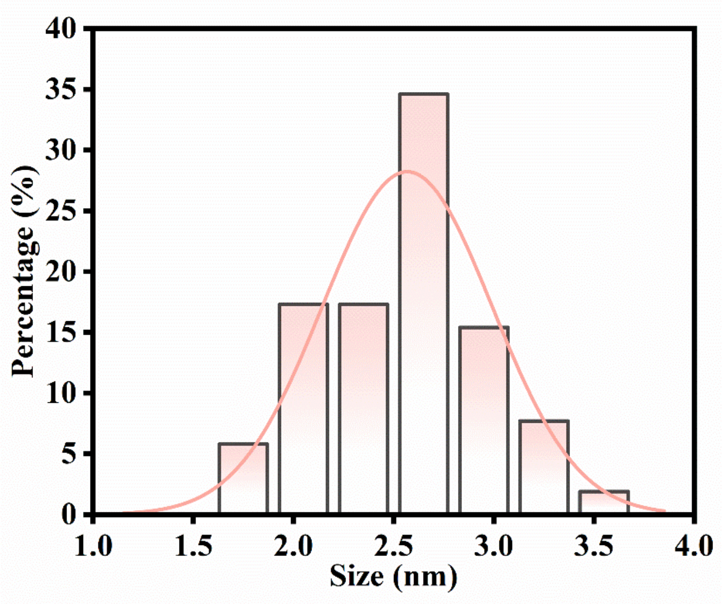

Figure S1. Size distribution of pristine CDs.

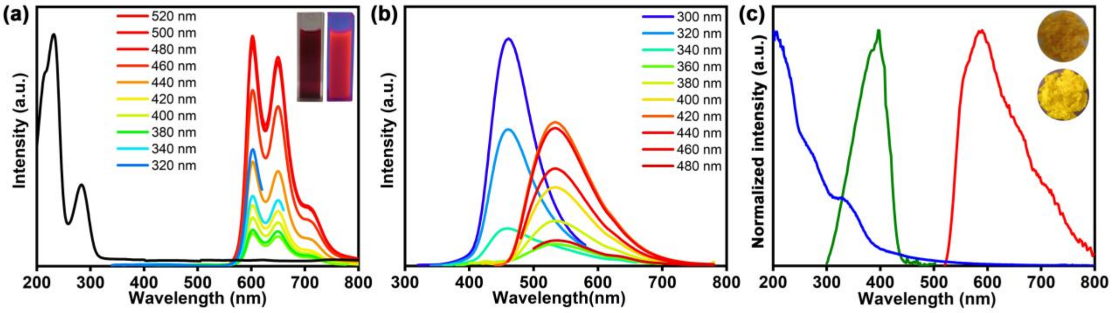

Figure S2. (a) Absorption and PL spectra at various excitation wavelengths for pristine CDs in ethanol. The inset shows the pristine CDs in ethanol under visible light (left) and 520 nm excitation (right). (b) PL spectra at various excitation wavelengths for ClSA-CDs in ethanol. (c) Absorption, excitation and PL spectra for ClSA-CDs in the solid-state. The inset shows photos of the ClSA-CDs in the solid-state under sunlight (top) and UV irradiation (bottom).

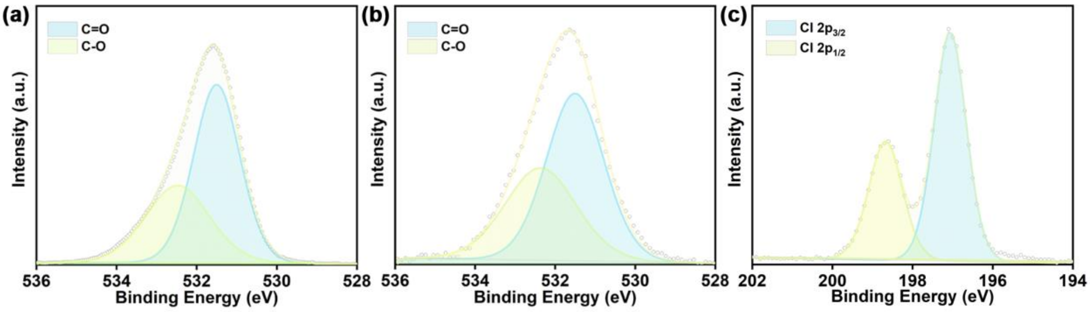

Figure S3. High-resolution O 1s XPS spectra for (a) CDs and (b) ClSA-CDs assemblies. (c) High-resolution Cl 2p XPS spectra for ClSA-CDs assemblies.

Table S1. Summarized XPS quantification data for the pristine CDs and ClSA-CDs assemblies.

Spectrum	Region	CDs	ClSA-CDs
Survey*	C (at. %)	49.77	76.29
	N (at. %)	13.17	2.91
	O (at. %)	37.05	16.77
	Cl (at. %)	/	4.04
C 1s	C-C/C=C (%)	59.26	65.55
	C-O/C-N (%)	36.11	27.34
	C=O/C=N (%)	4.64	7.11
N 1s	C=N (%)	69.32	77.08
	C-N (%)	30.68	22.92
O 1s	C=O (%)	62.54	59.51
	C-O (%)	37.46	40.49

* Atom percentages were calculated from peak areas of the C 1s, N 1s, O 1s and Cl 2p regions.

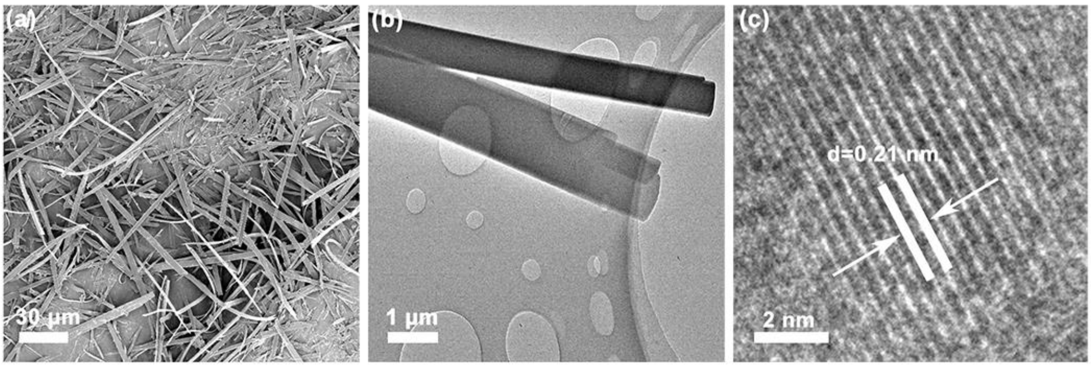

Figure S4. (a) SEM image, (b) TEM image and (c) HRTEM image of ClSA-CDs assemblies.

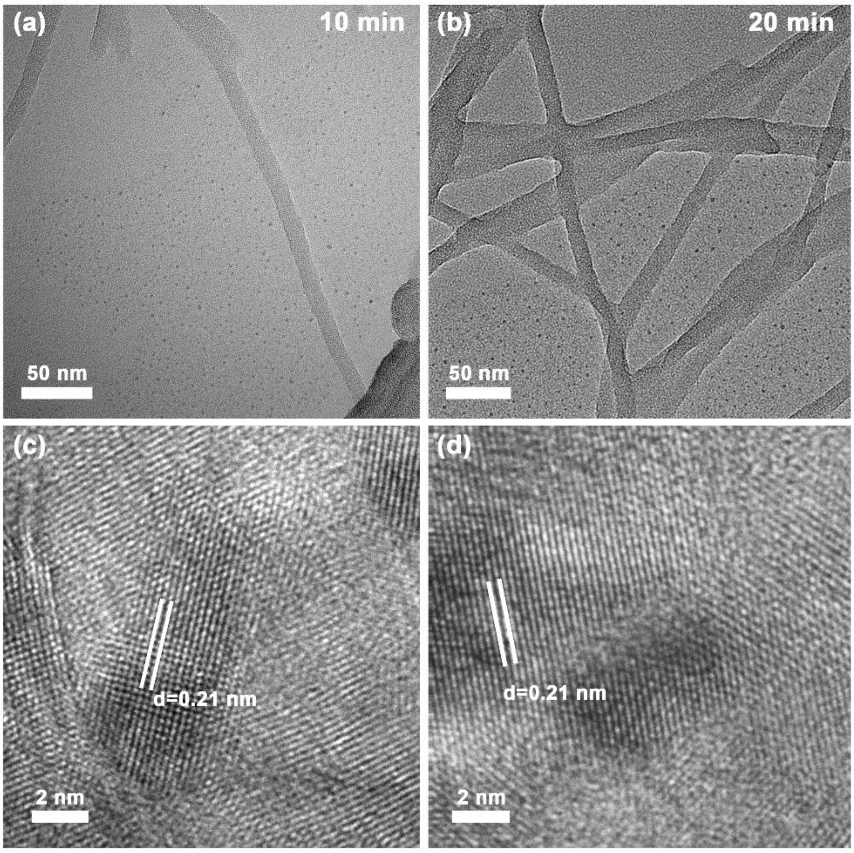

Figure S5. TEM and HRTEM images of pristine CDs heated with ClSA ligands at 80 ^{o}C for (a, c) 10 min and (b, d) 20 min.

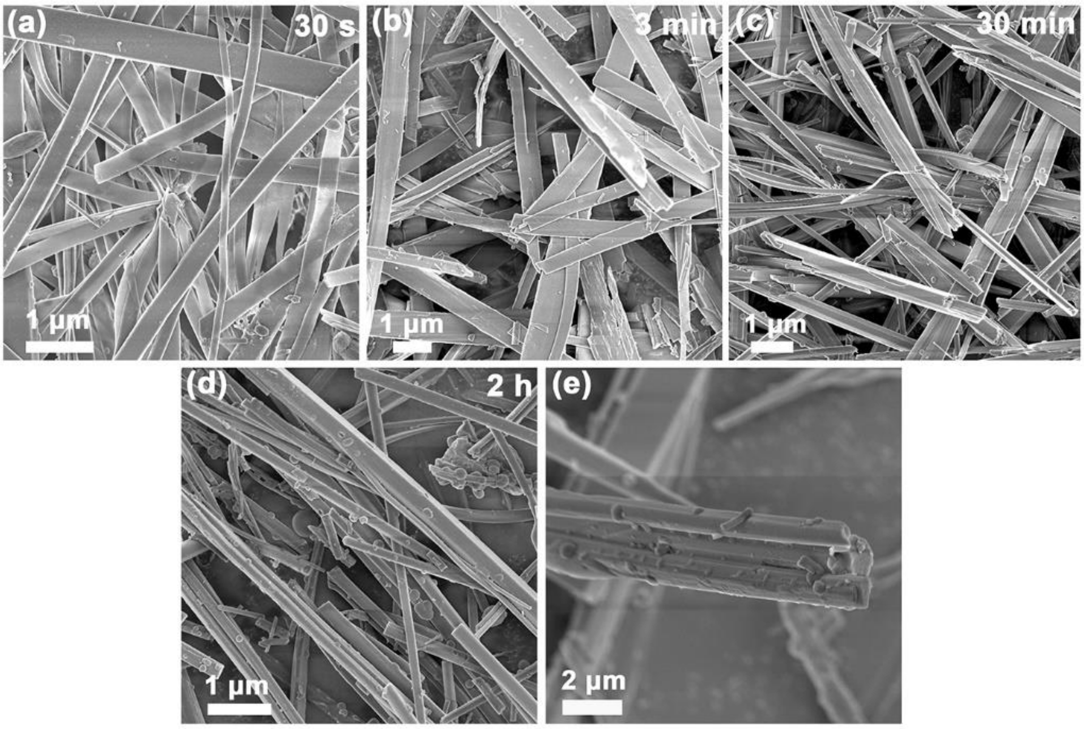

Figure S6. SEM images of assemblies of pristine CDs with CISA ligands heated at 80 °C for (a) 30 s, (b) 3 min, (c) 30 min and (d) 2 h. (e) Magnified image of the sample shown in (d).

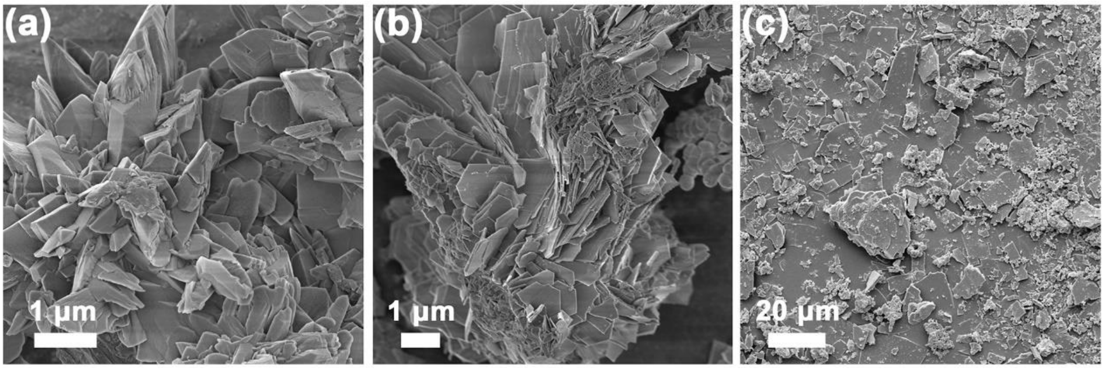

Figure S7. SEM images of assemblies of pristine CDs with (a) benzaldehyde ligands, (b) o-chlorobenzaldehyde ligands, or (c) salicylaldehyde ligands heated at 80 °C for 2 h.

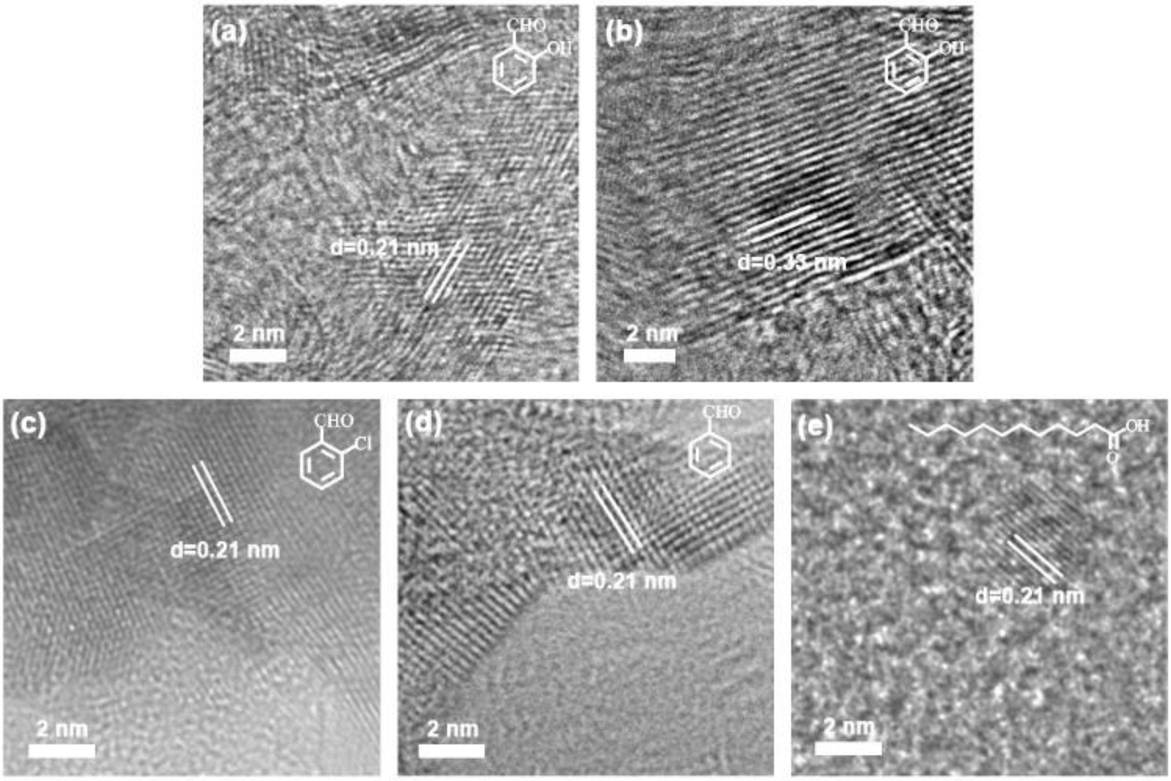

Figure S8. HRTEM images of pristine CDs heated with (a, b) salicylaldehyde ligands, (c) o-chlorobenzaldehyde ligands, (d) benzaldehyde ligands or (e) dodecanoic acid ligands at 80 °C for 2 h.

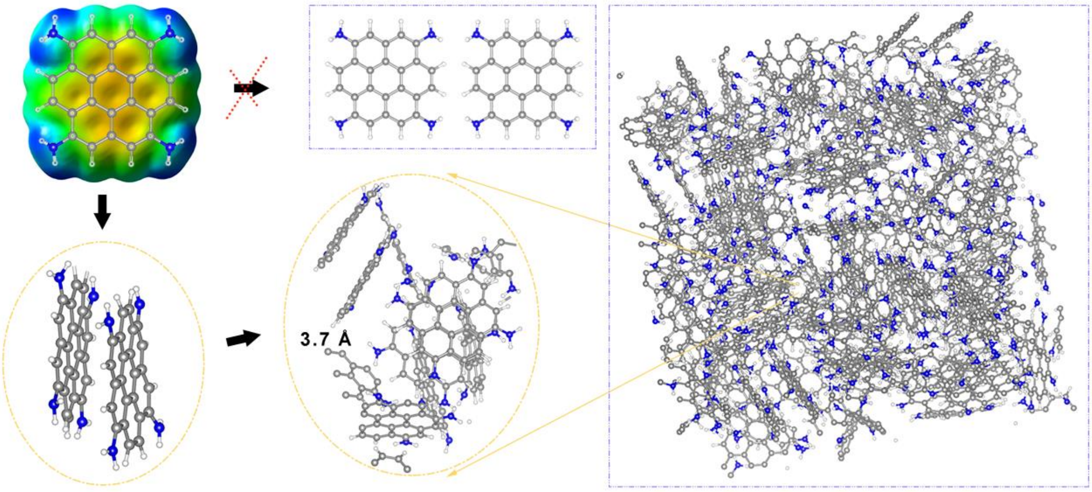

Figure S9. Random aggregation of pristine CDs via π-π interactions.

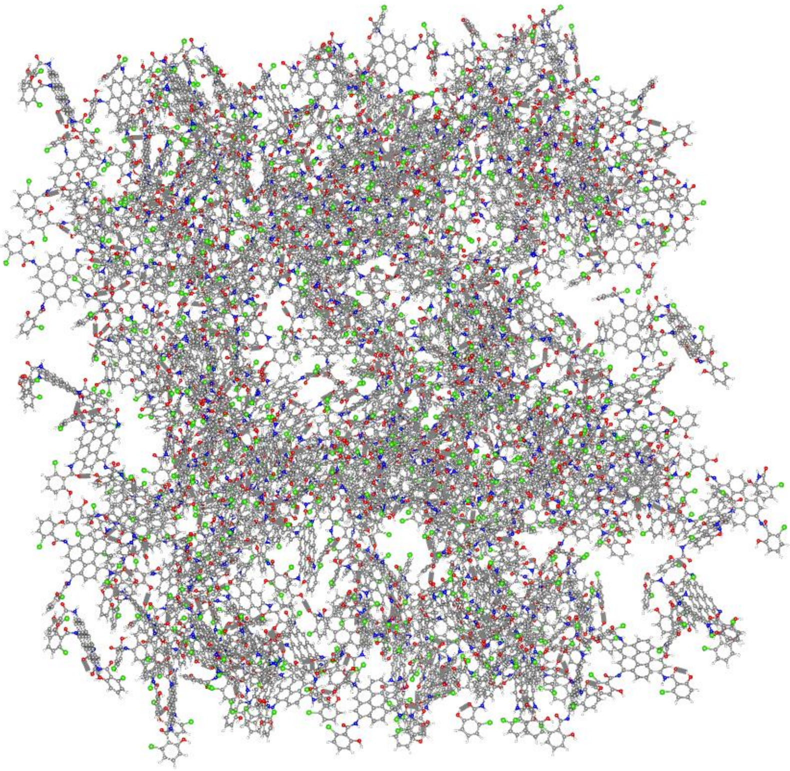

Figure S10. Ribbon-like assemblies formed by ligand-coated CDs.

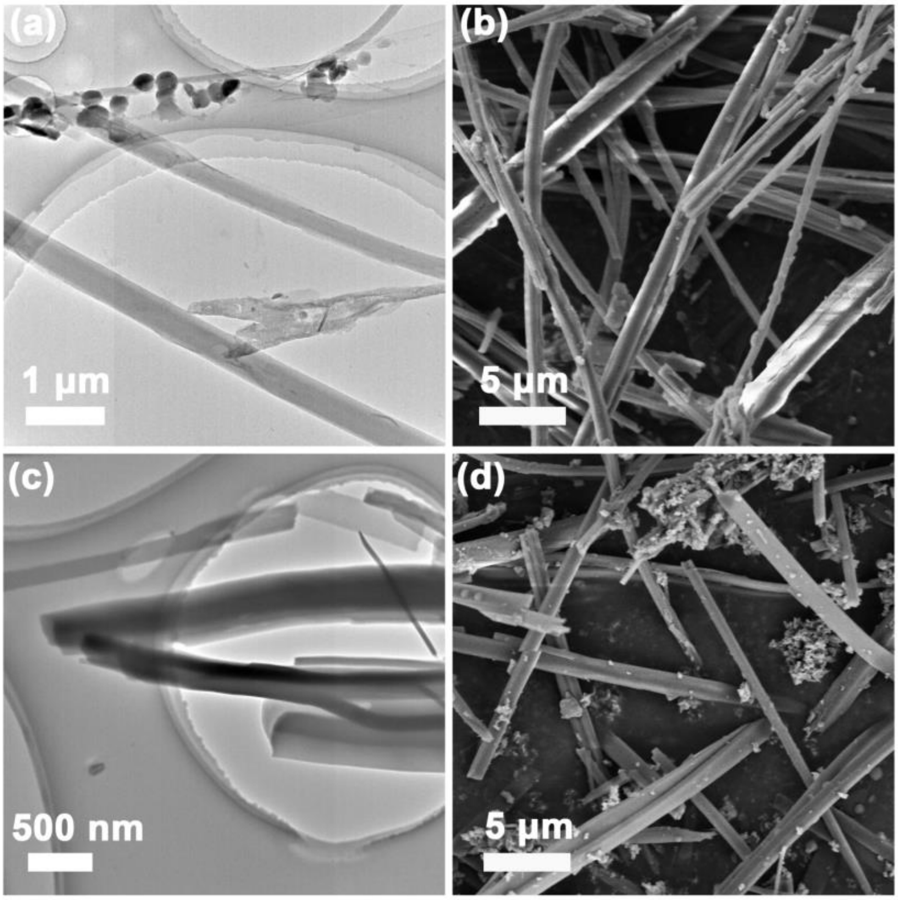

Figure S11. TEM and SEM images of assemblies of pristine CDs with ClSA ligands heated at (a, b) 50 ^{o}C for 2 h or (c, d) kept at room temperature.

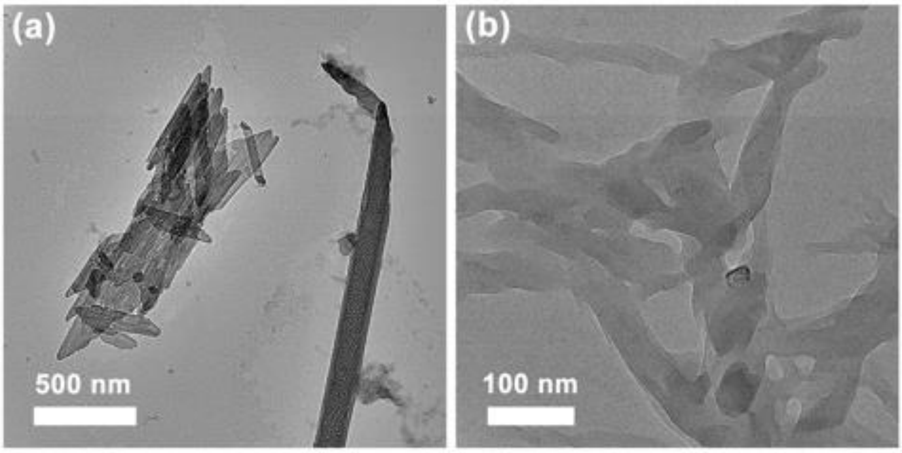

Figure S12. TEM images of assemblies of pristine CDs with insufficient ClSA ligands heated at 80 ^{o}C for 2 h.

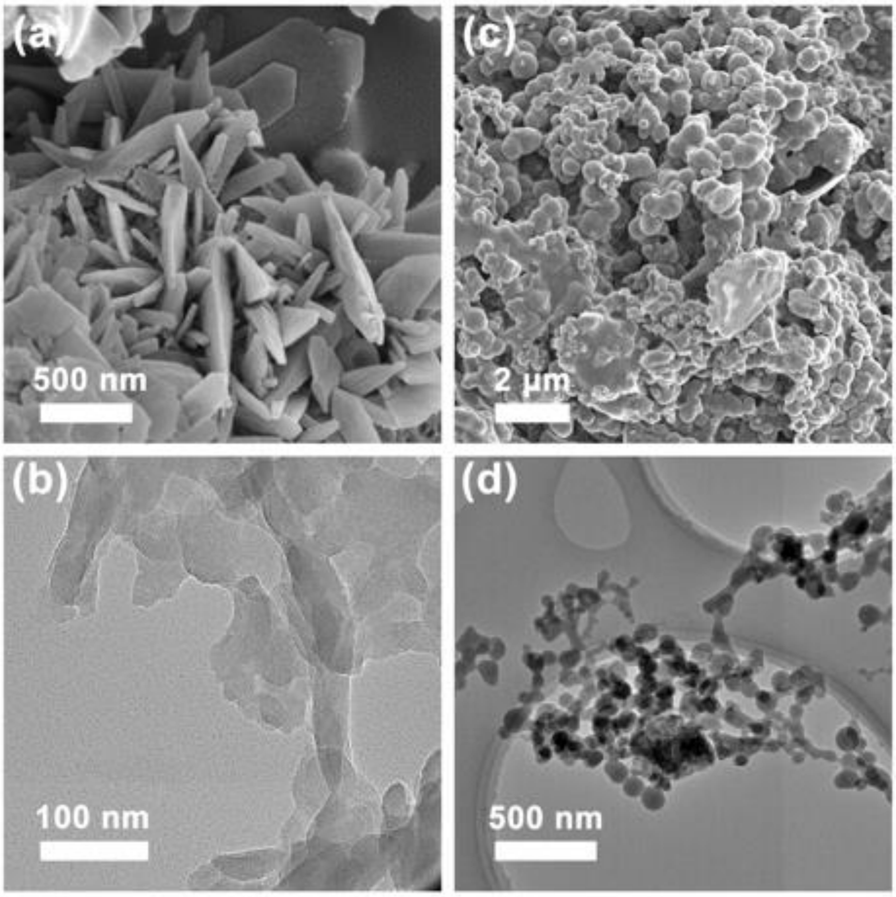

Figure S13. (a) SEM and (b) TEM images of assemblies of CDs2 with ClSA ligands heated at 80 ^{o}C for 2 h. (c) SEM and (d) TEM images of assemblies of CDs3 with ClSA ligands heated at 80 ^{o}C for 2 h.

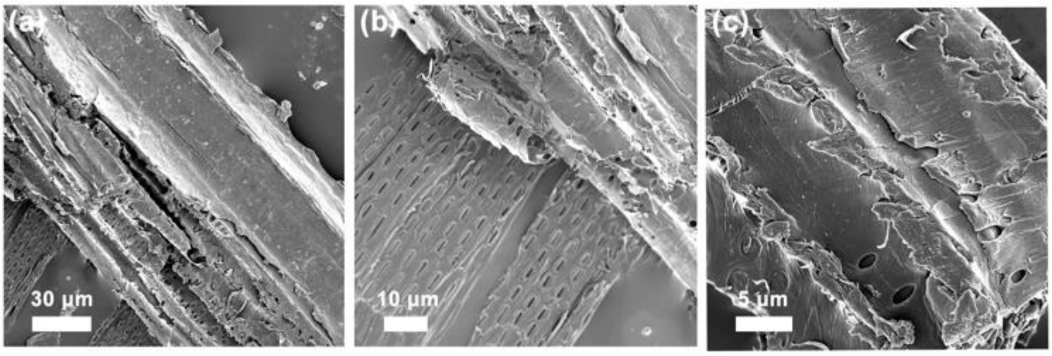

Figure S14. SEM images of ClSA ligands.

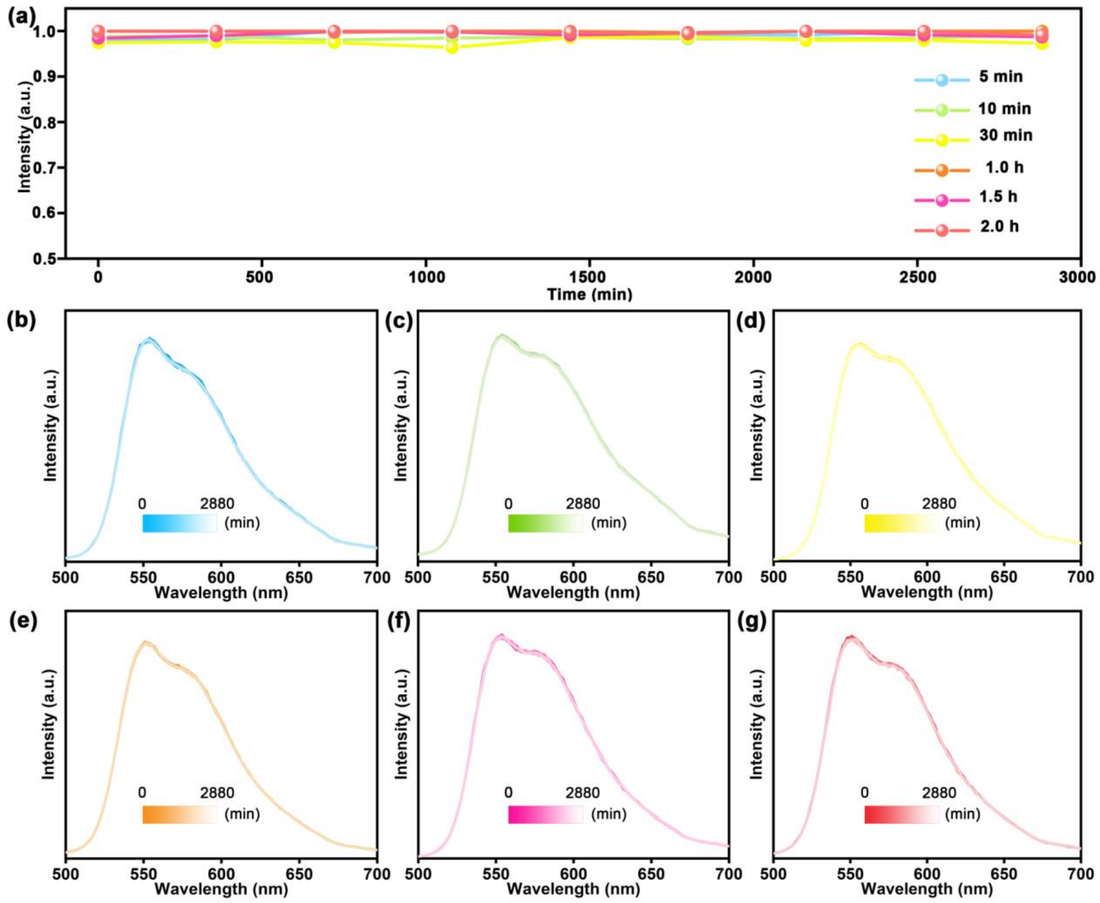

Figure S15. Stability of ClSA-CDs assemblies under UV (365 nm) irradiation for 2880 min. The stability experiment was conducted by irradiating the ClSA-CDs with a 365 nm hand-held UV lamp (WFH-204BS). The power of the UV lamp was 8 W.

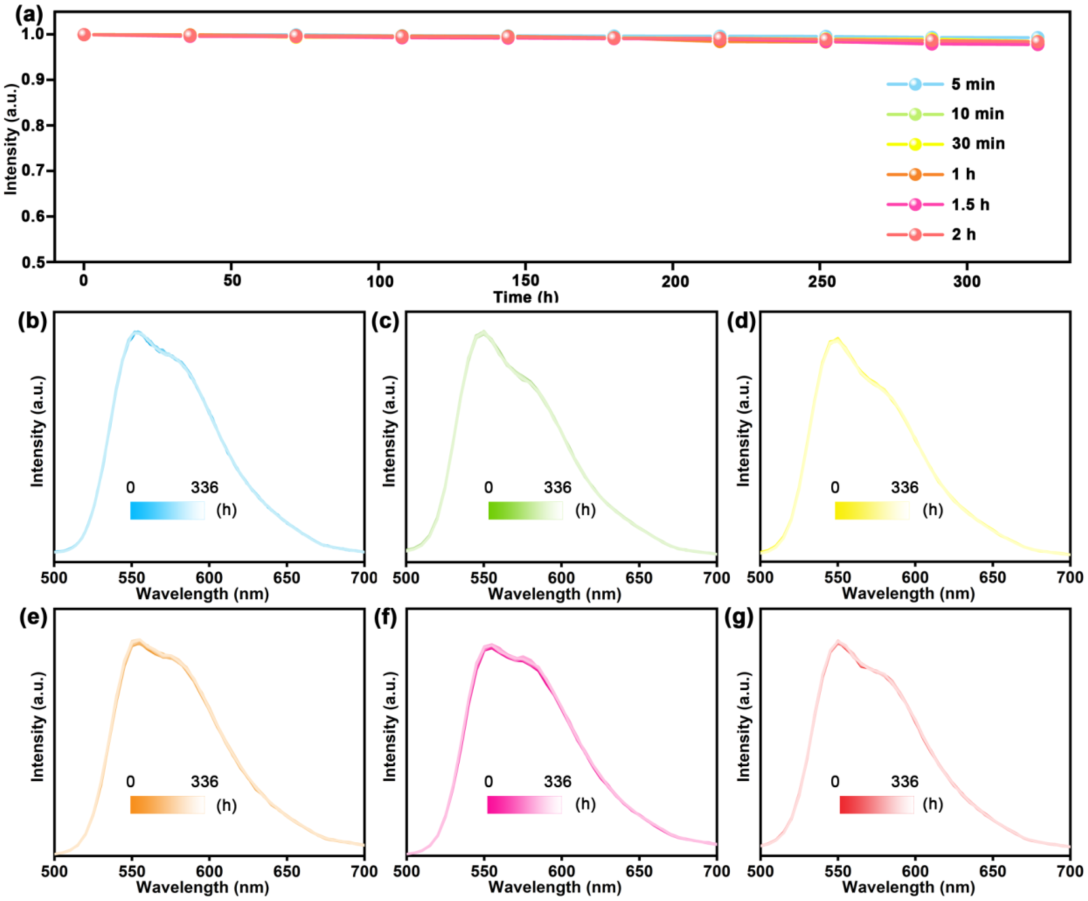

Figure S16. Stability of ClSA-CDs assemblies with heating at 80 ^{o}C in air for 336 h.

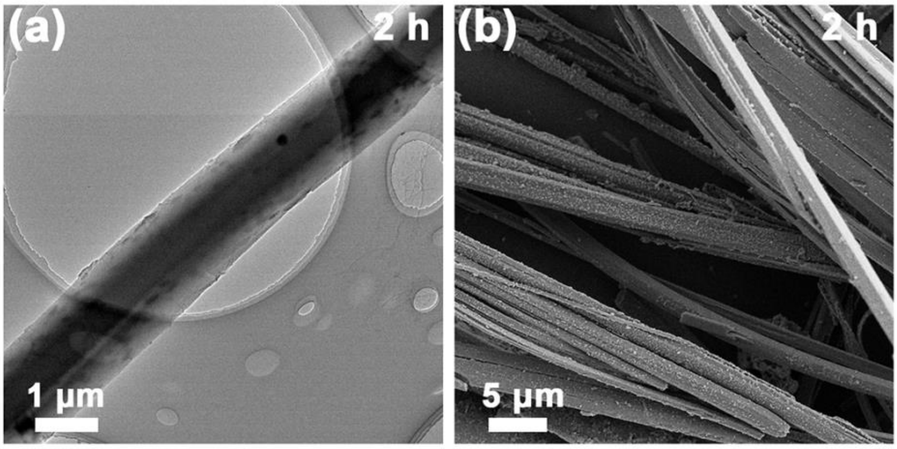

Figure S17. Morphology of ClSA-CDs assemblies after being left in an ambient environment for one year.

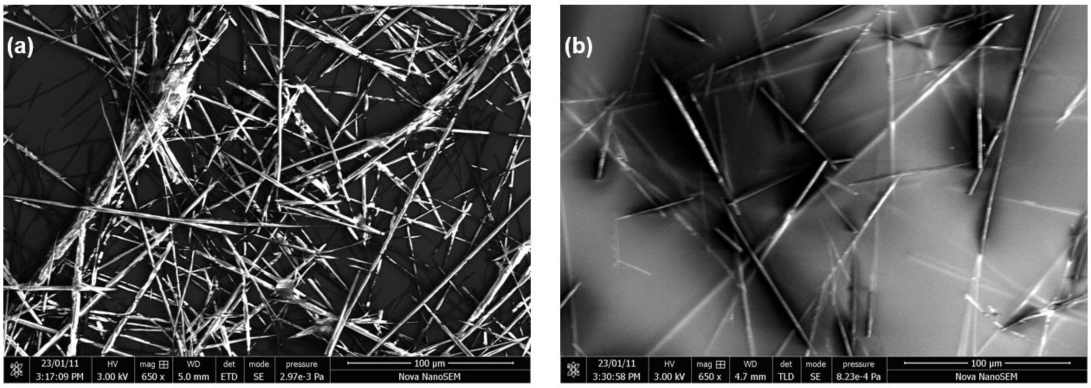

Figure S18. The SEM image of the film formed after dispersion of CDs aqueous solution and pss solution.

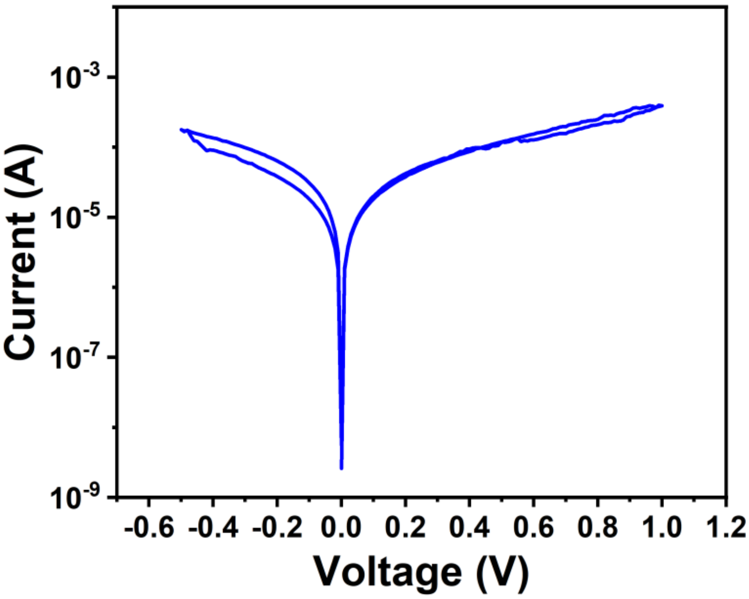

Figure S19. I-V curve of the pristine CDs-based memristor.

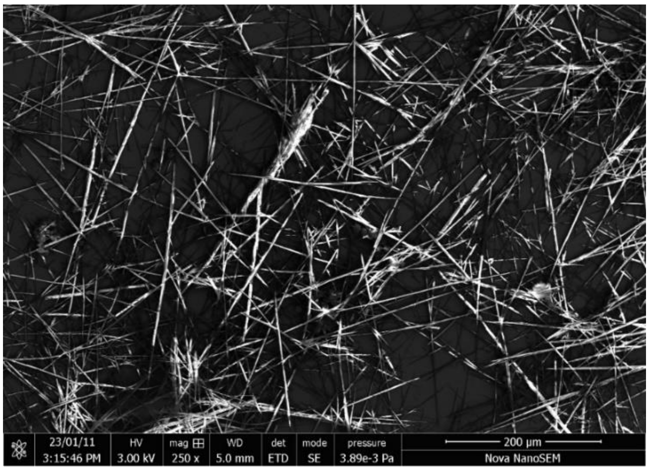

Figure S20. The distribution of ClSA-CDs in the device.

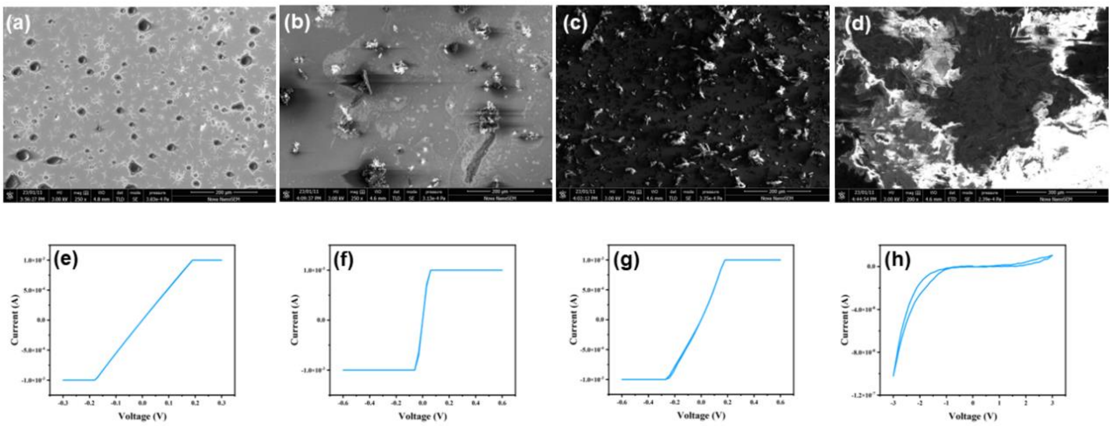

Figure S21. The dispersion of (a) benzaldehyde ligands, (b) o-chlorobenzaldehyde ligands, (c) salicylaldehyde ligands, and (d) dodecanoic acid ligands capped CDs in the device under the same experimental conditions. (e-h) I-V characteristic curve corresponding to a-d four materials respectively.

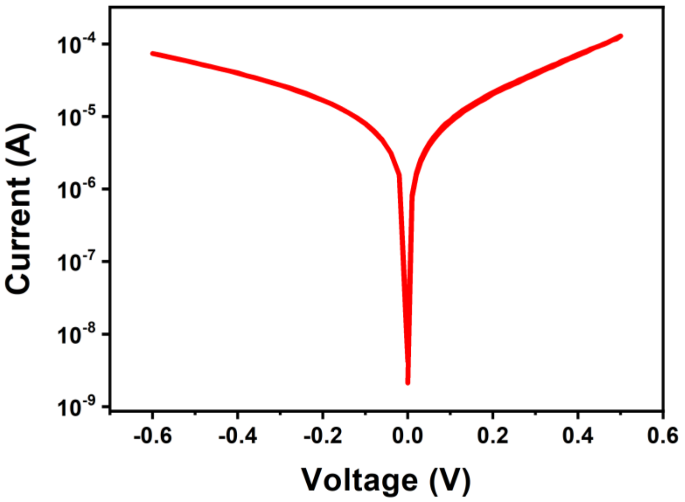

Figure S22. I-V curve of the ClSA ligand-based memristor.

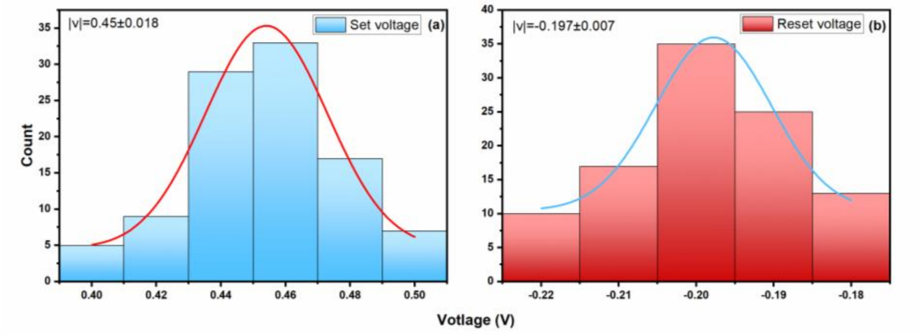

Figure S23. (a) The SET voltage distribution for ClSA-CDs devices. (b) The RESET voltage distribution for ClSA-CDs devices.

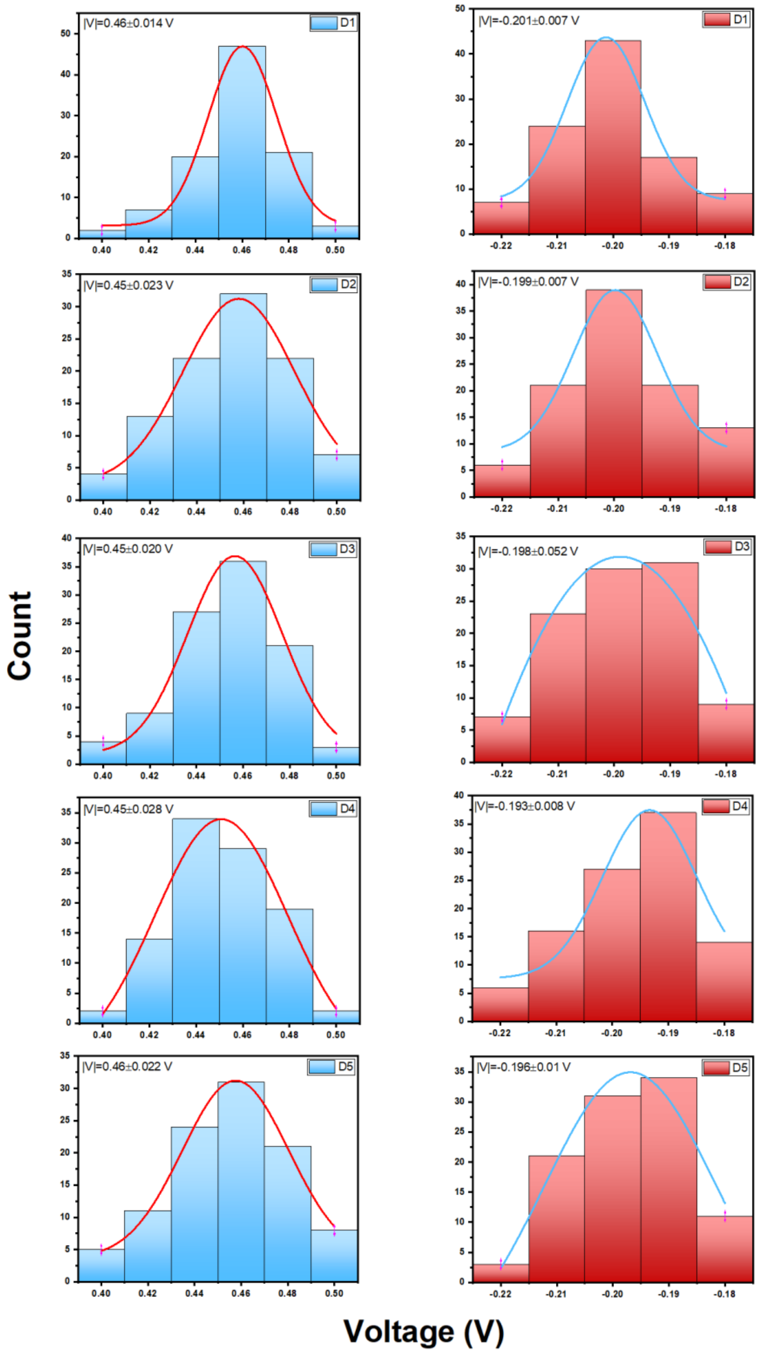

Figure S24. The threshold voltage distribution of five randomly selected devices.

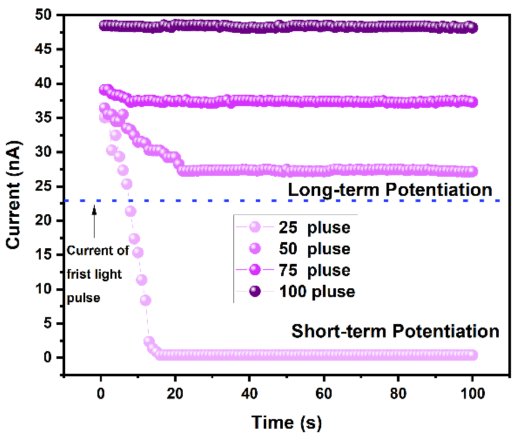

Figure S25. The current response after removing the light source after continuously applying 25, 50, 75 and 100 light pulses.

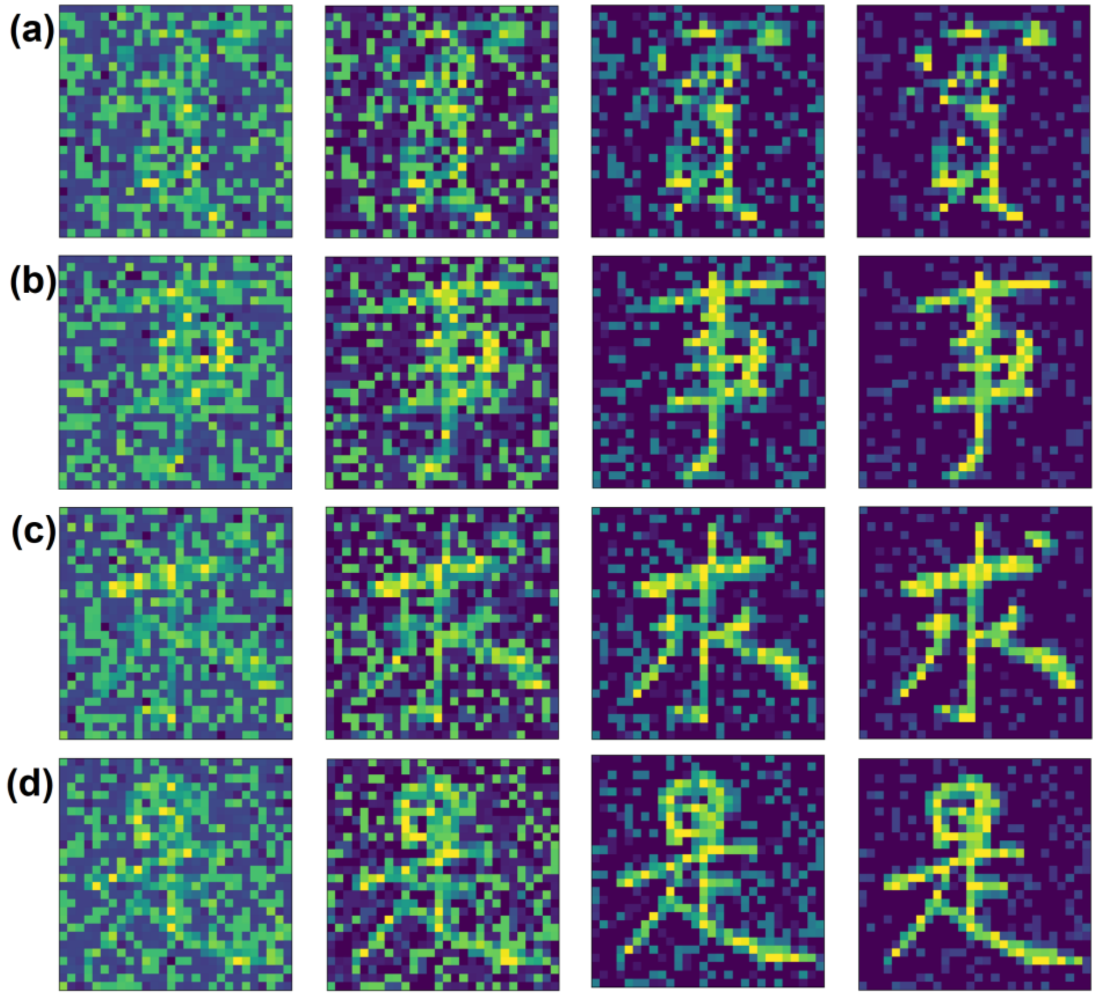

Figure S26. Chinese character image memory weight demonstration after 40, 80, 120, and 160 training iterations.

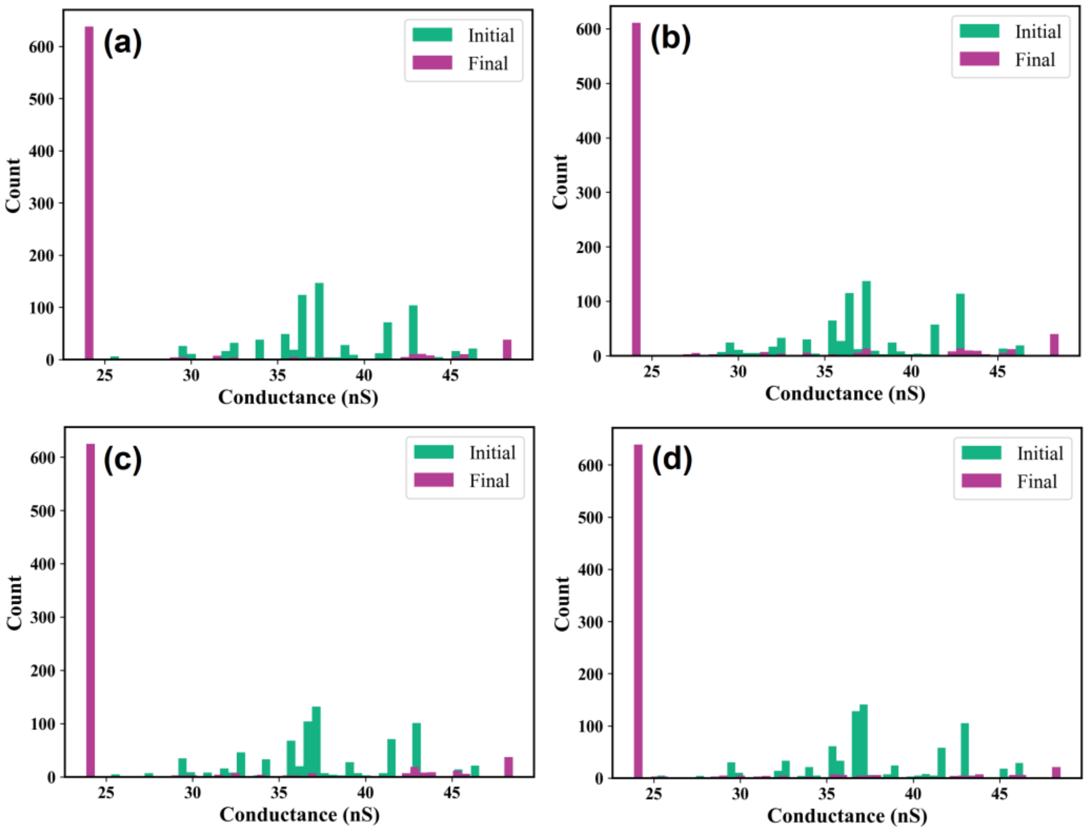

Figure S27. Distribution of synaptic weights for different Chinese characters: (a) 實, (b) 事, (c) 求, (d) 是.

Table S2. Characteristics of structurally different memristor devices based on 2D materials.

Structure	SET voltage (V)	RESET voltage (V)	Retention (s)	Optical device	Work temperature	Ref.
Pd/WS_2/Pt	0.6	-0.2	1.8×10^4	No	R. T.	Small 2019, 15, 1901423[1]
Gr/$MoS_{2-x}O_x$/Gr	1.0	-1	10^5	No	340 °C	Nat. Electron. 2018, 1, 130[2]
Ti/$MoTe_2$/Au	2.3	-1	10^3	No	R. T.	Nat. Mater. 2019, 18, 55[3]
Pd/MoO_x/ITO	2.5	-2.2	24 h	Yes	R. T.	Nat. Nanotechnol. 2019, 14, 776[4]
Au/Ti/h-BN/Au	~1.5	-0.6	N.A.	No	R. T.	Nat. Electron. 2018, 1, 458[5]
Ag/BiOI/Pt	0.05	-0.05	10^4	Yes	R. T.	Adv. Funct. Mater. 2022, 32, 2201276[6]
Al/WS_2/MoS_2/ITO	~1.2	~-1.75	5×10^3	No	R. T.	Nanoscale. 2021, 13, 11497[7]
Cu/MoS_2/Cu	~0.25	~-0.15	1.8×10^4	No	R. T.	Nano Lett. 2019, 19, 2411[8]
Ag/MoS_2/Pt	0.2–0.67	−1.5 ~−2.2	3 h	No	R. T.	NPJ 2D Mater. 2022, 6, 53[9]
Ag/MoO_x/MoS_2/Ag	~0.15	~-0.1	8×10^3	No	70 °C	Nat. Mater. 2015, 14, 199[10]
Au/MoS_2/Au	1.4	-0.7	1×10^4	No	R. T.	Nat. Commun. 2018, 9, 2524[11]
Au/MoS_2/Au	1.8	-1.1	N. A.	No	R. T.	Nat. Nanotechnol. 2021, 16, 58[12]
Au/MoS_2/Au	1.5	-1.5	1×10^4	No	R. T.	Nano Res. DOI: 10.1007/s12274-022-5042-7[13]
Ag/GeSe/Pt	6	-1.5	N. A.	No	R. T.	AIP Adv. 2020, 10, 045003[14]
Au/monolayer h-BN/Au	3	-1	2×10^4	No	R. T.	Adv. Mater. 2019, 31, 1806790[15]
Ag/hBN/Cu	0.72	−0.37	$\approx3\times10^3$	No	R. T.	Adv. Funct. Mater. 2016, 26, 2176[16]

Ag/h-BN/Graphene/ h-BN/Au	2.6	-4	10^6	No	R. T.	Nat. Commun. 2019, 10, 3161[17]
Au/ReS_2/hBN/Au	~10	~-15	2×10^3	No	R. T.	Nano Res. 2022, 15, 5443[18]
Al/GO/Al/PES	-2.5	2.5	6×10^4	No	R. T.	Nano Lett. 2010, 10, 4381[19]
Au/$HfSe_2$/Ti	0.6	~0.7	10^4	No	R. T.	Adv. Mater. 2022, 34, 2103376[20]
Ag/$MoSe_2$/Bi_2Se_3/ITO	0.68	-0.9	10^3	Yes	R.T.	Small. 2019, 15, 1805431[21]
Pt/$ReSe_2$/Pt	3.5	-0.75	2×10^4	No	R. T.	Adv. Mater. 2021, 33, e2007792[22]
TiN/C dots/ITO	0.4	-0.2	30 days	Yes	100 °C	This work

Table S3: Performance comparison with optoelectronic devices for visual learning.

**Functional layer**	**LTP/STP**	**Flexible**	**Fully transparent**	**Epoch times**	**Ref.**
ZnO	LTP	No	No	600	*Nano Energy,* 2021,89 106291
IGZO and the ion gel	STP	No	No	2000	*Adv. Funct. Mater.,* 2018, *28*, 1804397
MoO_x	LTP	No	No	1000	*Nat. Nanotechnol.,* 2019, *14* (8), 776-782
$CH_3NH_3PbI_3$	STP	No	No	4500	*Adv. Funct. Mater.*, 2018, 1806646
TiO_2:Ag	LTP	No	No	300	*Adv. Sci.*, 2022,9, 2104632
BFMO	STP	No	No	~3000	*Materials & Design*, 2022, 222,111046
ZnO/HfO_x	LTP	No	Yes	100	DOI: 10.1109/TED.2022.3233547
ion gel/GO/IGZO	STP	No	No	~10000	*Adv. Funct. Mater.* 2018, *28*, 1804397
CISA CD:pss	LTP	Yes	Yes	200	This work

Supplementary References

[1] X. Yan, Q. Zhao, A. P. Chen, J. Zhao, Z. Zhou, J. Wang, H. Wang, L. Zhang, X. Li, Z. Xiao, K. Wang, C. Qin, G. Wang, Y. Pei, H. Li, D. Ren, J. Chen, Q. Liu, *Small* **2019**, *15*, 1901423.

[2] M. Wang, S. Cai, C. Pan, C. Wang, X. Lian, Y. Zhuo, K. Xu, T. Cao, X. Pan, B. Wang, S.-J. Liang, J. J. Yang, P. Wang, F. Miao, *Nat. Electron.* **2018**, *1*, 130.

[3] F. Zhang, H. Zhang, S. Krylyuk, C. A. Milligan, Y. Zhu, D. Y. Zemlyanov, L. A. Bendersky, B. P. Burton, A. V. Davydov, J. Appenzeller, *Nat. Mater.* **2019**, *18*, 55.

[4] F. Zhou, Z. Zhou, J. Chen, T. H. Choy, J. Wang, N. Zhang, Z. Lin, S. Yu, J. Kang, H. S. P. Wong, Y. Chai, *Nat. Nanotechnol.* **2019**, *14*, 776.

[5] Y. Shi, X. Liang, B. Yuan, V. Chen, H. Li, F. Hui, Z. Yu, F. Yuan, E. Pop, H. S. P. Wong, M. Lanza, *Nat. Electron.* **2018**, *1*, 458.

[6] P. Lei, H. Duan, L. Qin, X. Wei, R. Tao, Z. Wang, F. Guo, M. Song, W. Jie, J. Hao, *Adv. Funct. Mater.* **2022**, *32*, 2201276.

[7] W. Zhang, H. Gao, C. Deng, T. Lv, S. Hu, H. Wu, S. Xue, Y. Tao, L. Deng, W. Xiong, *Nanoscale* **2021**, *13*, 11497.

[8] R. Xu, H. Jang, M.-H. Lee, D. Amanov, Y. Cho, H. Kim, S. Park, H.-j. Shin, D. Ham, *Nano Lett.* **2019**, *19*, 2411.

[9] M. Sun, M. Re Fiorentin, U. Schwingenschlögl, M. Palummo, *NPJ 2D Mater.* **2022**, *6*, 81.

[10] A. A. Bessonov, M. N. Kirikova, D. I. Petukhov, M. Allen, T. Ryhänen, M. J. A. Bailey, *Nat. Mater.* **2015**, *14*, 199.

[11] M. Kim, R. Ge, X. Wu, X. Lan, J. Tice, J. C. Lee, D. Akinwande, *Nat. Commun.* **2018**, *9*, 2524.

[12] J. Gigault, H. El Hadri, B. Nguyen, B. Grassl, L. Rowenczyk, N. Tufenkji, S. Feng, M. Wiesner, *Nat. Nanotechnol.* **2021**, *16*, 501.

[13] S. Wang, Z. Zhou, F. Yang, S. Chen, Q. Zhang, W. Xiong, Y. Qu, Z. Wang, C. Wang, Q. Liu, *Nano Res.*, DOI: 10.1007/s12274-022-5042-7.

[14] M. Zhao, R. Li, J. Xue, *AIP Adv.* **2020**, *10*, 045003.

[15] X. Wu, R. Ge, P.-A. Chen, H. Chou, Z. Zhang, Y. Zhang, S. Banerjee, M.-H. Chiang, J. C. Lee, D. Akinwande, *Adv. Mater.* **2019**, *31*, 1806790.

[16] K. Qian, R. Y. Tay, V. C. Nguyen, J. Wang, G. Cai, T. Chen, E. H. T. Teo, P. S. Lee, *Adv. Funct. Mater.* **2016**, *26*, 2176.

[17] L. Sun, Y. Zhang, G. Han, G. Hwang, J. Jiang, B. Joo, K. Watanabe, T. Taniguchi, Y.-M. Kim, W. J. Yu, B.-S. Kong, R. Zhao, H. Yang, *Nat. Commun.* **2019**, *10*, 3161.

[18] R. Li, D. Wang, *Nano Res.* **2022**, *15*, 6888.

[19] H. Y. Jeong, J. Y. Kim, J. W. Kim, J. O. Hwang, J.-E. Kim, J. Y. Lee, T. H. Yoon, B. J. Cho, S. O. Kim, R. S. Ruoff, S.-Y. Choi, *Nano Lett.* **2010**, *10*, 4381.

[20] S. Li, M.-E. Pam, Y. Li, L. Chen, Y.-C. Chien, X. Fong, D. Chi, K.-W. Ang, *Adv. Mater.* **2022**, *34*, 2103376.

[21] Y. Wang, J. Yang, Z. Wang, J. Chen, Q. Yang, Z. Lv, Y. Zhou, Y. Zhai, Z. Li, S.-T. Han, *Small* **2019**, *15*, 1805431.

[22] R. Ge, X. Wu, L. Liang, S. M. Hus, Y. Gu, E. Okogbue, H. Chou, J. Shi, Y. Zhang, S. K. Banerjee, Y. Jung, J. C. Lee, D. Akinwande, *Adv. Mater.* **2021**, *33*, 2007792.
